# Comparing stroke prevention therapy of direct oral anticoagulants and vitamin K antagonists in patients with atrial fibrillation: a nationwide retrospective observational study

**DOI:** 10.1186/s12916-020-01695-7

**Published:** 2020-08-27

**Authors:** Lena M. Paschke, Kerstin Klimke, Attila Altiner, Dominik von Stillfried, Maike Schulz

**Affiliations:** 1 Department of Prescription Data, Central Research Institute of Ambulatory Health Care in Germany, Salzufer 8, 10587 Berlin, Germany; 2 Department of General Practice, Medical Faculty, University of Rostock, 18055 Rostock, Germany

## Abstract

**Background:**

Direct oral anticoagulants (DOACs) are not only increasingly being used for the initial stroke prevention therapy but progressively also substitute vitamin K antagonist (VKA) treatment in patients with non-valvular atrial fibrillation (AF). DOACs have been compared regarding therapeutic efficacy and adverse outcomes to warfarin in several pivotal studies and showed non-inferiority in terms of stroke prevention and superiority in terms of bleeding complications. However, comprehensive comparative studies are lacking for phenprocoumon, a VKA prescribed frequently outside the USA and the UK and accounting for 99% of all VKA prescriptions in Germany. Patients treated with phenprocoumon seem to meet more often international normalized ratio values in the therapeutic range, which may have implications concerning their efficacy and safety. This study aims at comparing the risk of stroke and bleeding in phenprocoumon- and DOAC-treated patients with AF in an adequately powered observational study population.

**Methods:**

Retrospective analysis of stroke and bleeding incidence of 837,430 patients (1.27 million patient years) treated with DOAC or phenprocoumon for stroke prevention in German ambulatory care between 2010 and 2017. Relative risks of stroke and bleeding were estimated by calculating cox regression-derived hazard ratios (HR) and 95% confidence intervals (CI) of propensity score-matched cohorts.

**Results:**

Patients treated with DOAC had an overall higher risk for stroke (HR 1.32; CI 1.29–1.35) and a lower risk for bleeding (0.89; 0.88–0.90) compared to phenprocoumon. When analyzed separately, the risk for stroke was higher for dabigatran (1.93; 1.82–2.03), apixaban (1.52; 1.46–1.58), and rivaroxaban (1.13; 1.10–1.17) but not for edoxaban (0.88; 0.74–1.05). The risk for bleeding was lower for dabigatran (0.85; 0.83–0.88), apixaban (0.71; 0.70–0.73), and edoxaban (0.29; 0.17–0.51) but not for rivaroxaban (1.03; 1.01–1.04).

**Conclusions:**

This study provides a comprehensive view of the stroke and bleeding risks associated with phenprocoumon and DOAC use in Germany. Phenprocoumon may be preferable to DOAC treatment for the prevention of strokes in AF in a real-world population cared for in ambulatory care.

## Background

In 2011, the first direct oral anticoagulant (DOAC) was approved for stroke prevention in patients with atrial fibrillation (AF) [1, 2]. Since then, the number of DOAC prescriptions in Germany has steadily increased and since 2016 exceeded the number of previously preferred vitamin K antagonists (VKAs [3]).

While international, interventionally designed pivotal studies for DOACs found a reduced (dabigatran, apixaban) or similar (rivaroxaban, edoxaban) risk of stroke and systemic embolism, and a reduced (apixaban, edoxaban) or similar (dabigatran, rivaroxaban) risk of bleeding [4–7] compared to the VKA warfarin, practical experience and observational studies showed different effects of DOACs. Shortly after approval, evidence emerged of an increased risk of bleeding in patients treated with dabigatran [8], which has been attributed to an undesirable increase in plasma concentration mostly in patients with renal failure [9]. Due to continuing uncertainty about the risk of bleeding induced by DOACs, the European Medicines Agency (EMA) recently decided to review the results of an observational study commissioned by the EMA to determine whether changes to current conditions of use are required [10]. Other international observational studies investigated not only the safety but also the effectiveness of DOACs and showed similar risks for stroke, systemic embolism, and bleeding compared to warfarin, e.g., for dabigatran in a Danish cohort [11] and rivaroxaban in an American cohort [12].

However, these findings cannot directly be transferred to Germany and other countries, where phenprocoumon but not warfarin accounts for 99% of all VKA prescriptions. Phenprocoumon differs to warfarin in pharmacokinetic properties, such as metabolic mechanisms and plasma half-life, which lead to individual plasma concentrations [13]. The longer-acting phenprocoumon compared to the shorter-acting warfarin is associated with a higher proportion of time spent in the therapeutic range (TTR [14, 15]), which is why a direct comparison of phenprocoumon with DOACs is necessary.

The effectiveness and safety of DOACs compared to phenprocoumon in German population samples was recently investigated in three observational studies. Despite almost similar study designs and data sources, they showed completely or partially opposite results, with generally increased [16] or reduced [17] risks for stroke, embolism, and bleeding in DOACs, and for some DOACs an increased stroke risk but reduced bleeding risk [18]. One reason for these diverging findings might be the inclusion of different diagnoses to identify events of interest (e.g., inclusion [16] or exclusion [17] of pulmonary embolism in the endpoint “embolism”). In addition, these studies analyzed data from various (regional) health insurance funds (years 2011–2015), with 60,000 to 176,000 evaluable AF patients. Since the members of different German health insurance funds differ in baseline characteristics such as age, sex [19], and social status [20] and therefore also in overall morbidity, the different data sources could not only be responsible for deviating findings, but also pose the question of how representative the data are for the entire German population.

For this reason, the present observational study compared the risk of stroke, systemic embolism, and bleeding in AF patients treated with VKAs or DOACs by analyzing data from all statutory health insured (SHI) persons in Germany, who account for approximately 87% of the German population. In contrast to previous studies, the analysis of the prescriptions from 2011 to 2016 allows the inclusion of the DOAC edoxaban (approved in 2015), which, to our knowledge, has not yet been investigated in a Germany-related observational study.

## Methods

### Data source

The analyses are based on the nationwide ambulatory drug prescriptions data (AVD) and ambulatory billing claims data (VDA) of all residents with SHI in Germany (in 2016: approx. 71.4 million). They include prescriptions and diagnoses made by general practitioners and specialty doctors. Because the VDA are available quarterly (Additional file 1), linking the AVD and VDA allowed analyzing the prescribed medications and diagnoses of AF patients on a quarterly basis from the second quarter of 2010 to the fourth quarter of 2017 (study period).

### Study population

**Fig. 1:**
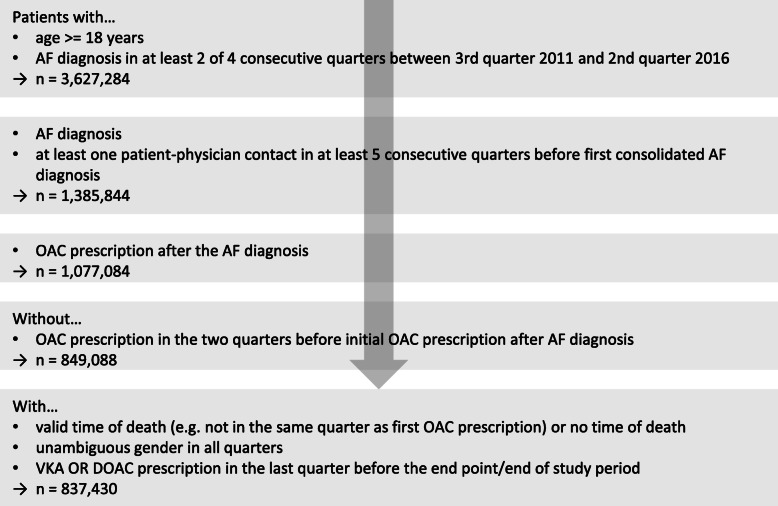
Definition of the study population. AF, atrial fibrillation; OAC, oral anticoagulant; VKA, vitamin K antagonist; DOAC, direct OAC; for final patient numbers of all study populations, see Table 2

The observational period started in the 3rd quarter of 2011 when the first DOAC was approved for patients with AF. For follow-up, data were available until the fourth quarter of 2016. After several steps (Fig. 1 and Additional file 1), the final study population included only patients with incident AF diagnoses and without previous prescriptions of oral anticoagulants (OACs). From the time of the initial OAC prescription, the patients were observed until an event of interest was diagnosed or the follow-up period ended.

### Endpoints and follow-up

In total, four primary endpoints were defined based on verified outpatient diagnoses (ICD-10-GM, Table S3) [21]. According to the indication of DOACs in patients with AF, the diagnosis of stroke, transient ischemic attack (TIA), systemic embolism, and non-traumatic bleeding represented an event of interest each. While stroke, TIA, and systemic embolism were defined by the corresponding, very specific ICD-10 codes, bleeding was defined by a composite of codes, representing intracerebral, extracerebral, and gastrointestinal minor and major bleeding events. Because the analyzed data provided no information about mortality, patients were considered dead if they had no physician contact in four consecutive quarters during the follow-up or post-follow-up period. Treatment discontinuation was defined by at last two consecutive quarters without OAC prescription. Patients who did not experience any primary endpoint during the follow-up period were censored in case of a treatment discontinuation, death, or end of the study period (Fig. S1). The endpoints were analyzed separately by building one cohort for each endpoint. As a consequence, the same patient could only be included once in each endpoint-related analysis, but with different endpoints than in the other cohorts. The survival time was calculated by subtracting the quarter of the OAC prescription from the quarter in which the event occurred or the patient was censored. Since the data were analyzed on a quarterly basis, the minimal time difference between the 1st OAC prescription and an event or censoring was one quarter (Additional file 1 and Table S1).

A high number of deaths were accompanied by treatment discontinuation. Therefore, in case of death, the criterion for treatment discontinuation was only met when patients did not receive any OAC prescription in two quarters before the defined period for death began (Fig. S1). When treatment discontinuation started only on quarter before death, the patient was classed as dead. However, as there was no clear marker for deaths in the data and the criteria for therapy discontinuation and death were not completely independent, the endpoint death was only evaluable with strict limitation (see also Additional file 1). For that reason, no hazard ratios but only incidence rates are reported for this endpoint, and the results need to be interpreted with caution.

### Treatment classification

According to the corresponding Anatomical Therapeutic Chemical (ATC) classification codes, prescriptions were defined as VKA (phenprocoumon, warfarin), dabigatran, rivaroxaban, apixaban, or edoxaban treatment (Table S2). In the general analysis, all DOACs were pooled as DOAC prescription. Patients with prescriptions of the same OAC over the entire follow-up period were classified as VKA- or DOAC-treated patients respectively, based on their first prescription. Patients with prescriptions of different OACs during follow-up, but only one type of OAC in their last quarter with OAC prescription were classified according to this last prescription. Additionally, they were tagged as switch users. Patients with more than one type of OAC prescription as their last prescription were excluded from the analysis, as an unambiguous classification was not possible. In the DOAC-specific analysis, each DOAC was analyzed separately. Patients were classified with treatment according to the ATC codes, and switch users were identified by prescriptions of different ATC codes.

The length of the follow-up depended on the occurrence of the individual endpoints. It was possible to switch between VKA and DOAC treatment between the first prescription of an OAC and the occurrence of an endpoint. For example, a patient may have changed from VKA to DOAC treatment after stroke and may not have experienced any other event of interest by the end of the study period. In this case, the patient was identified as a VKA user in the stroke-related study population. In all other study populations, this patient was identified as DOAC user according to his last OAC prescription before the end of the follow-up period. For this reason, some patients were classified in the endpoint-related study populations with different treatments, and each study population differed slightly in terms of patients included and treatment classification (Table 2).

### Statistical analysis

**Fig. 2:**
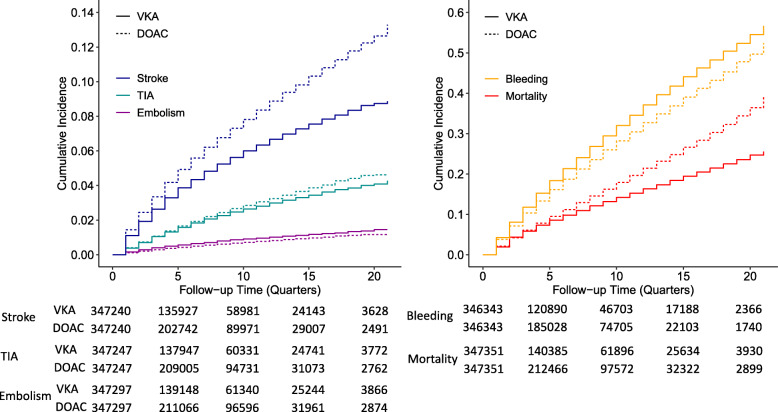
Cumulative incidence. VKA, vitamin K antagonist; DOAC, direct oral anticoagulant; TIA, transient ischemic attack; table indicates number of patients at risk. For number of censored patients, see Table S19 and S20

The survival times of VKA- and DOAC-treated patients were compared for each endpoint separately by calculating incidence rates (IR), hazard ratios (HR), and 95% confidence intervals (CI). Before the analysis, treatment groups were checked for balance in patient-related baseline characteristics like sex, age, and comorbidities (Table 1; for definitions of baseline variables, see Tables S4-S7). To assess the risk of stroke as accurately as possible, an individual CHA_2_DS_2_-VASc score [22] was calculated based on all available information about age, sex, and diagnosed pre-existing conditions (ICD-10 codes; Additional file 1). The individual comorbidity was assessed by using the Charlson comorbidity score [23] based on an algorithm for ICD codes (Additional file 1) [24–26]. To account for meaningful imbalances indicated by a standardized mean difference (SMD) of > 0.1 [27], a propensity score was calculated for each patient by performing a logistic regression, including the baseline variables, the CHA_2_DS_2_-VASc score, and the Charlson score. Finally, the score was used to form pairs of VKA- and DOAC-treated patients by using a nearest neighbor matching. Beginning with the VKA-treated patient with the largest propensity score, for each patient, the DOAC-treated patient with the closest propensity score was selected. To gain an optimal matching result, a caliper of 0.2 was used [28], that is, the propensity scores of each pair differed by at most 0.2 of the standard deviations of the propensity score. Each VKA-treated patient could be matched to one DOAC-treated patient only and vice versa. Matching was conducted separately for each endpoint-related study population for VKA vs. pooled DOACs as well as VKA vs. each DOAC. The final study populations were used to calculate HRs by using a multivariate Cox regression model [29]. Because switching between therapies may indicate an altered stroke and bleeding risk, the HR was adjusted for switching by including a binary variable as a covariate. The IR was calculated by dividing the number of new events by the total time under risk, experienced by the entire endpoint-related population. To allow a better comparison with other studies, the time under risk was recalculated from quarters into years and the IR was expressed per 1000 person years. For visualization, the cumulative incidence was calculated by dividing the number of events by the number of patients under risk for each quarter individually (Fig. 2).

**Table 1:** Patient characteristics

Stroke population after matching		VKA (*n* = 347,240)	DOAC (*n* = 347,240)	SMD	
				Before	After
Age	Mean(± SD)	75.78 (± 8.84)	75.70 (± 9.85)	0.03	0.01
	Median	77	77		
Age distribution (%)	18–36	0.09	0.19		
	37–54	2.14	3.22		
	55–72	27.53	27.43		
	73+	70.24	69.16		
Female sex (%)		52.02	53.00	− 0.07	− 0.02
CHA_2_DS_2_-VASc score	Mean (± SD)	4.45 (± 1.70)	4.39 (± 1.77)	0.12	0.04
	Median	4	4		
CHA_2_DS_2_-VASc score distribution (%)	0–1	3.77	5.29		
	2–3	25.12	25.28		
	4–5	44.74	43.23		
	6–7	22.41	22.02		
	8–9	3.96	4.18		
Charlson Comorbidity Index	Mean (± SD)	2.92 (± 2.63)	2.84 (± 2.63)	0.09	0.03
	Median	2	2		
Charlson Comorbidity Index distribution (%)	0–4	76.76	77.66		
	5–9	20.91	20.03		
	10–15	2.24	2.24		
	15+	0.16	0.14		
Number of distinct prescriptions (ATCs)	Mean (± SD)	12.50 (± 5.72)	12.43 (± 5.78)	0.05	0.01
	Median	12	11		
Number of prescriptions (ATCs)	Mean (± SD)	35.43 (± 21.17)	34.88 (± 21.43)	0.08	0.03
	Median	31	30		
Number of diagnoses (ICD codes)	Mean (± SD)	28.21 (± 15.39)	27.97 (± 15.52)	0.04	0.02
	Median	26	25		
Prescribed medicines (%)	Antiarrhythmic agents	90.47	90.41	0.01	0.00
	Antihypertensive drugs	80.94	80.39	0.06	0.01
	Antiplatelet drugs	25.14	25.56	− 0.03	− 0.01
	Corticosteroids (systemic use)	14.83	15.09	− 0.03	− 0.01
	Fondaparinux	0.78	0.68	0.02	0.01
	Heparins	27.02	23.44	0.36	0.07
	Insulin	10.98	10.57	0.04	0.01
	Lipid-lowering agents	47.61	46.35	0.09	0.03
	NSAIDs	40.00	40.79	− 0.05	− 0.02
	Oral anti-diabetic drugs	20.12	20.09	0.01	0.00
	Peptic ulcer/reflux disease	49.88	50.32	− 0.04	− 0.01
	SSRIs	5.39	5.62	− 0.04	− 0.01
Comorbidities (%)	Alcohol abuse/addiction	2.33	2.42	− 0.02	− 0.01
	Bleeding extracerebral	5.53	5.11	0.05	0.02
	Bleeding GI	5.81	5.68	0.02	0.01
	Bleeding intracerebral	0.45	0.48	− 0.02	0.00
	Cancer	19.77	19.80	0.00	0.00
	Coagulopathy	1.81	1.63	0.03	0.01
	Congestive heart failure	28.97	27.18	0.12	0.04
	COPD	16.04	15.79	0.02	0.01
	Coronary heart disease	40.67	38.72	0.13	0.04
	Diabetes	38.69	37.84	0.05	0.02
	Diverticulitis	10.66	10.80	− 0.01	0.00
	Embolism systemic	0.80	0.70	0.03	0.01
	Embolism venous	5.15	4.75	0.05	0.02
	Esophageal varices	0.21	0.21	0.00	0.00
	Hypertension	88.14	87.79	0.05	0.01
	Ischemic stroke	8.62	8.41	0.02	0.01
	Liver disease	13.48	13.31	0.02	0.00
	Nicotine use/dependence	4.44	4.51	− 0.01	0.00
	Renal disease	14.88	13.31	0.12	0.04
	Upper GI	25.97	26.25	− 0.03	− 0.01
	Vascular disease	21.99	21.54	0.04	0.01
	Vascular dementia	1.87	1.99	− 0.05	− 0.01
	Venous malformation	1.85	1.64	0.04	0.02

*VKA* vitamin K antagonist, *DOAC* direct oral anticoagulant, *SMD* standardized mean difference, *SD* standard deviation, *ATCs* Anatomical Therapeutic Chemical classification codes

For all statistical tests, only balanced datasets were used and an alpha level of *α* = 0.05 was considered as significant. Since the study was explorative, no correction was applied for multiple comparisons. The analyses were conducted with R [30] (for specific packages see Additional file 1) [31–37].

### Sensitivity analysis

To describe a full picture of all patients with AF and VKA or DOAC prescription, broadly defined criteria in the general analysis included patients independent of the received dose (approved or not approved for AF, see also Additional file 1) [38, 39] and independent of alternative diagnoses, which also might have indicated a DOAC prescription. Subpopulations excluding patients with dabigatran or rivaroxaban prescriptions of not approved doses or with alternative diagnoses are analyzed in sensitivity analyses 1 and 2 (for exclusion criteria, see Tables S8-S9).

## Results

In total, 837,430 patients with AF fulfilled the inclusion criteria and received VKA (412,406) or DOAC (434,767) treatment (multiple treatment classifications per patient were possible, see the “Methods” section). Exact numbers of endpoint-related study population are shown in Table 2. The separate study populations of the individual DOACs included 55,131 dabigatran, 234,802 rivaroxaban, 133,970 apixaban, and 14,666 edoxaban users.

**Table 2:** Number of patient populations

		**Number of patients before matching**					
		**VKA**	**DOAC**	**Dabigatran**	**Rivaroxaban**	**Apixaban**	**Edoxaban**
Study population	Stroke	405,437	430,940	53,057	228,609	131,751	14,276
	Embolism	404,823	431,816	52,796	228,522	132,872	14,556
	TIA	405,012	431,574	52,839	228,593	132,547	14,442
	Bleeding	408,402	427,477	53,233	229,934	127,613	13,266
	Mortality	404,695	431,978	52,763	228,499	133,059	14,586
		**Number of patients after matching**					
		**VKA**	**DOAC**	Dabigatran*	Rivaroxaban*	Apixaban*	Edoxaban*
Study population	Stroke	347,240	347,240	53,057	228,600	131,748	14,276
	Embolism	347,297	347,297	52,796	228,513	132,869	14,556
	TIA	347,247	347,247	52,839	228,584	132,544	14,442
	Bleeding	346,343	346,343	53,233	229,926	127,610	13,266
	Mortality	347,351	347,351	52,763	228,490	133,056	14,586

*VKA* vitamin K antagonist, *DOAC* direct oral anticoagulant, *TIA* transient ischemic attack

*Number of VKA patients equals number of DOAC patients

Before matching, the VKA vs. DOAC populations differed in the mean CHA_2_DS_2_-VASc score (exemplary numbers for stroke-related study population: VKA = 4.48 vs. DOAC = 4.28), the proportion of patients with prescribed heparins (36.44% vs. 18.91%) and the proportion of patients with congestive heart failure (28.92% vs. 27.19%), coronary heart disease (40.56% vs. 38.76%), and renal disease (14.85% vs. 13.32%; Tables S10-S14). The difference in heparin prescriptions was presumably due to an initial concomitant therapy in VKA users to bridge a first phase of higher coagulation risk (Additional file 1) [40]. When the patients were compared separately according to their DOAC prescription, the mean CHA_2_DS_2_-VASc score, the median age, and the proportion of female patients were highest in apixaban users (Table S22). In total, 86,954 patients were assigned as switch users, because they had at least one DOAC prescription after an initial VKA prescriptions (*n* = 60,521) or vice versa (*n* = 27,733).

After matching, on average 85.6% VKA and 80.6% DOAC patients remained in the study populations, including 55,131 dabigatran, 234,794 rivaroxaban, 133,969 apixaban, and 14,666 edoxaban users (see also Table 2). In the matched datasets, an SMD of < 0.1 SD for all variables indicated no remaining meaningful imbalance (Tables 1 and S15-S18). The mean follow-up time was 5.00 quarters for VKA and 6.71 quarters for DOAC patients. On average, 14.9% of final DOAC and 6.9% of final VKA users had switched between OAC treatments during follow-up, and in 64.8% VKA and 21.5% DOAC users, a treatment discontinuation was identified.

In total, 31,048 strokes, 11,925 TIAs, 3596 systemic embolism, 115,536 bleeding events, and 72,211 deaths occurred. When comparing the follow-up time of VKA und DOAC users, adjusted HRs indicated higher risks for stroke (HR 1.32; 95% CI 1.29–1.35) and TIA (1.10; 1.06–1.14) and lower risks for systemic embolism (0.78; 0.73–0.83) and bleedings (0.89; 0.88–0.90) in DOAC users (Table 3). The cumulative incidence was higher for stroke, TIA, and mortality and lower for embolism and bleeding in DOAC than in VKA users (Table 3 and Fig. 2).

**Table 3:** Results of joint DOAC vs. VKA

Events	Incidence rate per 1000 PY		Adjusted HR (95% CI)	*p* value
	VKA	DOAC		
Stroke	26.69	33.45	1.32 (1.29–1.35)	< .001
TIA	11.16	11.71	1.10 (1.06–1.14)	< .001
Embolism (systemic)	4.01	2.99	0.78 (0.73–0.83)	< .001
Bleeding	136.57	117.7	0.89 (0.88–0.90)	< .001
Mortality (all cause)	61.36	73.5	–	–

*VKA* vitamin K antagonist, *DOAC* direct oral anticoagulant, *TIA* transient ischemic attack, *PY* person years, *HR* hazard ratio, *CI* confidence interval

Compared to VKA users, the risk for stroke and TIA was highest in dabigatran users (stroke: 1.93; 1.82–2.03, TIA: 1.33; 1.22–1.45), followed by apixaban (stroke: 1.52; 1.46–1.58, TIA: 1.15; 1.08–1.22) and rivaroxaban (stroke: 1.13; 1.10–1.17, TIA: 1.06; 1.01–1.11; Table 4). For edoxaban, no significant difference in risk for stroke (0.88; 0.74–1.05) but a reduced risk for TIAs (0.71; 0.53–0.95) was observed. The risk for systemic embolism did not differ between VKA and dabigatran users (0.93; 0.79–1.10), but was lower in rivaroxaban (0.83; 0.77–0.90), apixaban (0.75; 0.67–0.85), and edoxaban (0.29; 0.17–0.51) compared to VKA users. The bleeding risk was lower in patients treated with dabigatran (0.85; 0.83–0.88), apixaban (0.71; 0.70–0.73), or edoxaban (0.74; 0.68–0.81) than in patients treated with VKA. Rivaroxaban users showed slightly increased risks for bleedings compared to VKA users (1.03; 1.01–1.04; Table 3).

**Table 4:** Results of separate DOAC vs. VKA

Events	Incidence rate per 1000 PY		Adjusted HR (95% CI)	*p* value
	VKA	Dabigatran		
Stroke	26.77	45.94	1.93 (1.82–2.03)	< .001
TIA	11.64	14.13	1.33 (1.22–1.45)	< .001
Embolism (systemic)	3.69	3.07	0.93 (0.79–1.10)	.42
Bleeding	134.82	107.77	0.85 (0.83–0.88)	< .001
Mortality (all cause)	52.34	63.49	–	–
	VKA	Rivaroxaban		
Stroke	25.88	27.77	1.13 (1.10–1.17)	< .001
TIA	10.69	10.83	1.06 (1.01–1.11)	.02
Embolism (systemic)	3.79	2.99	0.83 (0.77–0.90)	< .001
Bleeding	135.19	133.23	1.03 (1.01–1.04)	< .001
Mortality (all cause)	57.79	75.02	–	–
	VKA	Apixaban		
Stroke	27.58	38.81	1.52 (1.46–1.58)	< .001
TIA	11.58	12.33	1.15 (1.08–1.22)	< .001
Embolism (systemic)	3.77	2.75	0.75 (0.67–0.85)	< .001
Bleeding	137.8	93.36	0.71 (0.70–0.73)	< .001
Mortality (all cause)	64.26	74.45	–	–
	VKA	Edoxaban		
Stroke	26.24	15.53	0.88 (0.74–1.05)	.16
TIA	11.18	5.18	0.71 (0.53–0.95)	.02
Embolism (systemic)	3.94	1.31	0.29 (0.17–0.51)	< .001
Bleeding	133.92	69.82	0.74 (0.68–0.81)	< .001
Mortality (all cause)	53.58	30.62	–	–

*VKA* vitamin K antagonist, *DOAC* direct oral anticoagulant, *TIA* transient ischemic attack, *PY* person years, *HR* hazard ratio, *CI* confidence interval

### Sensitivity analysis

In the first sensitivity analysis, 7629 patients with rivaroxaban or dabigatran prescriptions in doses that are not approved for stroke prevention in AF patients were excluded from the population. After exclusion, all matched populations were still balanced (all SMD < 0.1). When comparing VKA and DOAC users, the HRs for all endpoints where similar to the main results (Table S23). Analyzing rivaroxaban and dabigatran separately revealed similar numerical HRs as in the main analysis, with still weaker increased risk for TIA and bleeding in rivaroxaban users (Table S23). In the second sensitivity analysis, 26,810 patients with additional diagnoses to AF, which might have also indicated a DOAC prescription, were excluded from the analysis, when the diagnosis took place in the two quarters before or in the same quarter of the first OAC prescription. After exclusion, the matched populations were still balanced (all SMD < 0.1) and all results were similar to the main results (Table S24).

## Discussion

### Joint DOAC compared to VKA

The present study compares the relative risks of stroke, TIA, systemic embolism, and bleeding in patients with AF who have been prescribed either DOAC or VKA, based on a population of all residents in Germany with SHI in 2011–2016. After successful matching, the populations were considered as comparable regarding the included patient characteristics, whereby only confounders could be considered for which data were available (see the “Limitations” section). The findings show a generally increased risk of stroke and TIA as well as higher IR for mortality in DOAC users than in VKA users. The only Germany-related study, which jointly compared all DOAC users to VKA users, found similar effects for stroke, TIA, and mortality [16]. Deviating from Müller et al. [16], the present study revealed a generally lower risk of systemic embolism and bleeding in DOAC, compared to VKA users. For the endpoint systemic embolism, these differences might result from different inclusion criteria: In contrast to Müller et al. [16], the present study did not consider pulmonary embolisms in the analysis, as in patients with AF, DOACs are approved for prevention of stroke and systemic embolism but not for pulmonary embolism. Also, with regard to bleeding events, both studies differ. To capture all bleeding events which might be related to the pharmaceutical therapy, the present study comprehensively included non-traumatic bleeding events regardless of their location, while Müller et al. [16] included gastrointestinal bleedings and respiratory bleedings only. Nevertheless, similar directions for the relative risk of bleeding could have been expected. More similar to the present results, a meta-analysis of the pivotal studies of all DOACs showed lower risks of major bleeding in DOAC users compared to warfarin users [41]. In addition, two Germany-related studies that analyzed the risk of bleeding for each DOAC separately found lower risk of bleeding in apixaban and dabigatran users and a slightly increased [17] or similar [18] risk in rivaroxaban users, which also supports the present results.

### Separate DOAC compared to VKA

#### Stroke, TIA, and systemic embolism

When analyzing the DOACs separately, an increased risk of stroke is found for dabigatran, rivaroxaban, and apixaban compared to VKAs. While Ujeyl et al. [18] also observed an increased risk of stroke for apixaban, the risk of stroke in dabigatran and rivaroxaban compared to VKA users was only non-significantly increased. This difference may be due to the lower statistical power of the previous study, as the present study analyzed 2–4 times the number of patients. In contrast, Hohenloser et al. [17] found a decreased risk of stroke in dabigatran and apixaban and no difference in rivaroxaban compared to VKA users. Similarly, also, the pivotal study of dabigatran found a decreased risk of stroke compared to warfarin [4], but all other DOACs showed no difference compared to warfarin [5–7]. The discrepancies between the pivotal studies and the present results may be caused by the different VKAs that have been used. While the TTR of patients treated with warfarin was 55–65% in the pivotal studies, the TTR in the German population (with 99% phenprocoumon prescriptions) can be assumed to be between 68 and 79% [14, 42]. A prolonged TTR can be expected to be associated with a reduced risk of stroke and as a consequence might result in a beneficial stroke prevention therapy compared to DOACs. Further, differences between the studies could stem from general methodological differences, such as inclusion criteria, which were broader in the present study. On the one hand, this procedure may allow less specific conclusions for individual subgroups of patients; on the other hand, the results may be more representative of the patients who actually receive OAC treatment. For instance, all pivotal studies excluded patients with an estimated creatinine clearance (CrCl) < 30 ml/min or < 25 ml/min (apixaban), while the present study does not differentiate between CrCl-levels. In patients with impaired renal function, considering an individual adjustment of the dose is recommended for all DOACs [38, 39, 43, 44]. While high concentrations of DOAC in serum increase the risk of bleeding [9], low doses increase the risk of stroke compared to high doses [4–7]. Especially for dabigatran, the uncertainty about an increased risk of bleeding shortly after approval [8], and particularly in patients with renal impairment [9], may have led to its cautious use, so that lower dosages rather than higher dosages tend to be prescribed. This approach could entail an increased risk of stroke, as found in the present study. A recent retrospective American study supports this explanatory approach: Shpak et al. [45] also found higher stroke incidences among overall DOAC compared to warfarin users. When considered individually, this effect was strongest for apixaban and dabigatran, whereas rivaroxaban showed only marginally and edoxaban no significantly increased stroke risk. In combination with reduced stroke and elevated intracranial bleeding risk in patients with strong anticoagulation, the authors see the results as an indication that DOAC patients may be less strongly anticoagulated than VKA patients. Because an easy-to-use test to evaluate the blood coagulability in DOAC users is lacking, and all DOAC have shorter half-lives than VKAs (5–17 h vs. 24–130 h), fluctuations in anticoagulation are more likely to occur with DOACs than with VKAs. Further investigations, in which patients with low and high DOAC doses are analyzed separately, are needed to reveal the reasons behind the increased risk of stroke in DOAC users. Unfortunately, the available data do not provide any information about clinical parameters that would indicate a justified dose reduction (such as CrCl or body weight). Furthermore, it is important to emphasize that the VKA and DOAC populations could only be adjusted as accurately as the available data allowed (see the “Limitations” section). Therefore, prospective studies are needed to further compare the effect of DOAC and VKA treatment in patients with impaired renal function.

Parallel to an increased stroke risk, DOAC users show the highest risk of a TIA when they received dabigatran, followed by apixaban and rivaroxaban. But the risk for systemic embolism is similar (dabigatran) or lower (rivaroxaban, apixaban, and edoxaban) in DOAC compared to VKA users. This is surprising as it may be assumed that the mechanisms behind strokes, TIA, and systemic embolisms caused by AF largely overlap. Thus, the same OAC should have similar effects on all these events. Notable, in the present study, the incidence of systemic embolism was much lower than the incidence of all other endpoints, leading to lower statistical power. As we still found a significant reduction in risk of embolism, the result may imply differences in the pathogenesis of strokes and systemic embolisms caused by AF.

Interestingly, edoxaban was the only DOAC to not show an increased but a similar risk of stroke and decreased risks of TIA and systemic embolism compared to VKAs. Similarly to apixaban and rivaroxaban, edoxaban acts by inhibiting the factor Xa and should therefore be expected to have comparable effects than other factor Xa inhibitors. In relation to edoxaban, but not apixaban and rivaroxaban, there have been concerns about low concentrations in serum, that is why the U.S. Food and Drug Administration (FDA) does not recommend edoxaban in AF patients with a CrCl > 95 ml/min [46]. High CrCl may lead to reduced efficacy of edoxaban. Even though in Germany no recommendations in relation to high CrCl levels have been published, the knowledge about the American conditions of use may have been influential. In case edoxaban is associated with rather low serum concentrations, higher doses may be more likely to be prescribed than lower doses. It is also conceivable that edoxaban is preferentially prescribed in patients with renal impairment, who can be assumed to have generally higher DOAC serum concentrations than patients without renal impairment. At least 20% of 70–79-year-old Germans has a renal impairment due to their age [47]. Both cases would lead to more efficient prevention of stroke, TIA, and embolism (as observed in the present study). However, the patient characteristics in the present study show that—with 13%— the percentage of patients with renal impairment is moderate and only the second highest in edoxaban users after apixaban user (15%), which partly contradicts the latter theory. Noteworthy, because in the present study, edoxaban is the latest DOAC approved in 2015, its users make up the smallest study population with the shortest follow-up period of max. 1 year. Therefore, the results should be interpreted with caution and future studies are necessary, to further investigate the observed differences between DOACs.

#### Bleeding and mortality

All DOACs but rivaroxaban showed a reduced risk of bleeding, which corresponds with the findings of Germany-related and international observational studies [11, 12, 17, 18, 48, 49]. Also, the pivotal studies of all DOAC showed lower (apixaban, edoxaban) or at least similar (dabigatran, rivaroxaban) risks of major bleeding in DOAC compared to warfarin users [4–7]. Edoxaban also showed a reduced risk of bleeding but was not yet analyzed by any Germany-related study or in comparison to the VKA phenprocoumon. The present bleeding-related results are in line with findings from the pivotal [5] and international studies [50].

Because the data provided no explicit information about mortality and similar criteria were used to determine therapy discontinuation and time of death (specific number of consecutive quarters without prescription/diagnosis), no statistical tests or HR but only IR are reported. The IR for mortality showed higher rates for dabigatran, rivaroxaban, and apixaban compared to VKA users, which is in line with higher IR for DOACs overall [16]. In contrast, Ujeyl et al. [18] and Hohnloser et al. [17] reported similar or lower IR for mortality in dabigatran users. Edoxaban showed lower IR for mortality while the pivotal study found no difference in risk [5]. The divergent findings underline the need to interpret the present results related to mortality with caution.

#### General

The results of the main analysis are supported by the sensitivity analyses, which indicate that the inclusion criteria were selected carefully and that all patients included in the analysis were AF patients who received OAC prescription for stroke prevention. Further, the inclusion of all doses in the analysis had no significant influence on the main results.

The therapy discontinuation in VKA users was much higher compared to DOAC users, which is well-known from previous international and Germany-related studies [16, 51, 52]. Similar to Müller et al. [16], in most cases, the therapy was discontinued early after only a few prescriptions, which explains the shorter mean follow-up time for VKA than for DOAC users. By censoring patients with therapy discontinuation, the present study accounted for this phenomenon.

Although the patient adherence could not be analyzed with the present data, it can be expected that the risk of an occurring event also depends on the regular intake of OACs. In line with the assumption that less frequently needed intake of tablets simplifies being adherent, several studies indicate that a once-daily treatment is associated with better treatment success than twice-daily treatment [53, 54]. In the present study, a similar pattern is observable, as dabigatran and apixaban user show a higher stroke risk compared to VKA user than rivaroxaban and edoxaban, which require a once-daily administration. On the contrary, there might be evidence that the risk of bleeding is lower in case of two-daily treatment because the dose is distributed throughout the day [54]. Taken together, the expected patient’s adherence should be considered in the decision for a specific OAC.

### Limitations

The present study has several limitations. Importantly, the analyzed data are restricted to outpatient diagnoses and do not include any information from the inpatient sector. This means that patients, who are hospitalized after experiencing an event of interest, can only be observed at the time a doctor in the outpatient sector codes the relevant event. Therefore, the length of follow-up time may be prolonged after the event of interest actually occurred. As this applies to all patients independently of the OAC therapy, this should not bias the comparison between VKAs and DOACs. Further, patients who die in hospital after an event of interest can only be identified as deaths. Information on the diagnosis that led to hospitalization is missing, which results in an underestimation of the number of events actually occurring. Therefore, the absolute IR and HR numbers need to be treated with caution. However, since the relative number of events and most results are consistent with literature, the outpatient data should contain a reliable number of relevant diagnoses.

Also determined by the underlying data, the survival times were calculated based on quarters. Thus, an identification of survival time differences between patients with VKA and DOAC treatment is limited to a minimum of one quarter (see Table S1).

As in all studies that analyze observational data, the patient populations with DOAC and VKA treatment may still differ (marginally) in some of the considered characteristics despite the successful matching procedure. Further, limited adjustment was possible for characteristics such as alcohol consumption and smoking, which can be expected to be only diagnosed and coded in serious cases, while data of the regular consumption are missing. Similarly, no clinical measures were available to exactly adjust for hypertension (e.g., blood pressure), renal impairment (e.g., CrCl levels), and overweight (e.g., bodyweight). Also no socioeconomic data regarding income, education, and occupation group or data on ethnicity were available, which are known to influence the general health-related behavior [55]. However, the advantage of the present study is its large, representative dataset.

## Conclusions

By analyzing a large proportion of all VKA and DOAC users with AF, the present study shows that VKA and DOAC users differ in their relative risks of stroke and bleeding and that these differences vary slightly depending on the individual DOAC therapy. These findings support the recommendation that the decision for VKA or DOAC treatment should depend on the patient’s individual stroke and bleeding risk [56]. In the case of DOAC treatment, the same applies to the selection of the specific DOAC. The present results provide additional information to help decide which treatment is more appropriate for which patient. Furthermore, patients’ preferences should be addressed when deciding on the therapy for stroke prevention in patients with AF. This analysis strongly supports that a routine substitution with a DOAC for patients on stable VKA therapy is not appropriate.

The results presented here are, at least in part, consistent with the results of comparable studies done before. However, further confirmatory randomized controlled trials are necessary to determine the efficiency and safety of DOACs. This applies especially to patient groups that predominantly receive DOACs, such as elderly and or patients with renal impairment.

## Supplementary information

**Additional file 1.** Study design details, description of study variables, patient characteristics before and after matching, sensitivity analyses.

## Data Availability

The data were analyzed with the permission of the regional Associations of Statutory Health Insurance Physicians in Germany (ASHIP). The authors are not permitted to make the data publicly available because of the highly confidential nature of ambulatory drug prescriptions and billing claims data. Permission to access the data is restricted to researchers and subject to the consent of ASHIPs.

## Abbreviations

AF: Atrial fibrillation
ATC: Anatomical Therapeutic Chemical
CI: Confidence interval
CrCl: Creatinine clearance
DOAC: Direct oral anticoagulant
HR: Hazard ratio
INR: International normalized ratio
VKA: Vitamin K antagonist
