# Supplementary material for: Comparing stroke prevention therapy of direct oral anticoagulants and vitamin K antagonists in patients with atrial fibrillation: a nationwide retrospective observational study
Source: BMC Med. 2020 Aug 27;18:254. doi: 10.1186/s12916-020-01695-7 (PMC7450597; doi:10.1186/s12916-020-01695-7)
Supplement: Supplementary file 1 — Additional file 1. Study design details, description of study variables, patient characteristics before and after matching, sensitivity analyses. [file 12916_2020_1695_MOESM1_ESM.docx]

Additional file to: Comparing stroke prevention therapy of direct oral anticoagulants and vitamin-K-antagonists in patients with atrial fibrillation: a nationwide retrospective observational study

Lena M Paschke^1^, Kerstin Klimke^1^, Attila Altiner^2^, Dominik von Stillfried^1^, Maike Schulz^1^

^1^ Department of Prescription Data, Central Research Institute of Ambulatory Health Care in Germany, Salzufer 8, 10587 Berlin / Germany

^2^ Department of General Practice, Medical Faculty, University of Rostock, 18055 Rostock / Germany

Table of contents

[1 Methods 2](#_Toc43362355)

[1.1 Data source 2](#_Toc43362356)

[1.2 Study Population 2](#_Toc43362357)

[1.3 Endpoints and Follow-up 3](#_Toc43362358)

[1.4 Treatment classification 5](#_Toc43362359)

[1.5 Measurements 5](#_Toc43362360)

[1.5.1 Events of Interest 5](#_Toc43362361)

[1.5.2 CHA_2_DS_2_-VASc Score 6](#_Toc43362362)

[1.5.3 Charlson Comorbidity Score 7](#_Toc43362363)

[1.5.4 Comorbidities: Medication 7](#_Toc43362364)

[1.5.5 Comorbidities: Diagnoses 8](#_Toc43362365)

[1.5.6 Sensitivity analysis I: Definition of alternative diagnoses 8](#_Toc43362366)

[1.5.7 Sensitivity analysis II: Definition of approved dosages 9](#_Toc43362367)

[1.6 R-Packages 9](#_Toc43362368)

[2 Results 9](#_Toc43362369)

[2.1 Joint DOAC Analysis 9](#_Toc43362370)

[2.1.1 Patient Characteristics before matching 9](#_Toc43362371)

[2.1.1.1 Stroke Population 9](#_Toc43362372)

[2.1.1.2 TIA Population 11](#_Toc43362373)

[2.1.1.3 Embolism Population 12](#_Toc43362374)

[2.1.1.4 Bleeding Population 13](#_Toc43362375)

[2.1.1.5 Mortality Population 14](#_Toc43362376)

[2.1.2 Patient Characteristics after matching 15](#_Toc43362377)

[2.1.2.1 TIA Population 15](#_Toc43362378)

[2.1.2.2 Embolism Population 17](#_Toc43362379)

[2.1.2.3 Bleeding Population 18](#_Toc43362380)

[2.1.2.4 Mortality Population 19](#_Toc43362381)

[2.2 Cumulative incidence 20](#_Toc43362382)

[2.3 Heparin related Analysis 22](#_Toc43362383)

[2.4 Separate DOAC Analysis 23](#_Toc43362384)

[2.4.1 Patient characteristics before matching: Stroke Population 23](#_Toc43362385)

[2.4.2 Sensitivity Analysis I and II 25](#_Toc43362386)

# Methods

## Data source

The analyses are based on the nationwide ambulatory drug prescriptions data (AVD) and ambulatory billing claims data (VDA) of all residents with SHI in Germany (in 2016: approx. 71.4 million). According to §300 (2) SGB V, the AVD are provided by the data processing centers for pharmacies by order of the regional Associations of Statutory Health Insurance Physicians (ASHIPs) and include information about outpatient prescriptions, which were redeemed in a pharmacy. According to §295 SGB V, the VDA are provided by the ASHIPs directly and include personal data (e.g. sex, age, residential area) as well as the diagnoses of all outpatients who have contacted a physician at least once a year. While the AVD are available on a daily basis, the date of the VDA diagnoses is limited to yearly quarters. Linking the AVD and VDA using patient information allowed analyzing the prescribed medications and diagnoses of AF-patients on a quarterly basis. Including a pre- and post-observation phase, the entire dataset covered the period from the 2nd quarter of 2010 to the 4th quarter of 2017.

## Study Population

Defining the final study populations involved several steps (see also Figure 1). First, patients with a verified AF diagnosis (ICD-10 Code I48) in at least two out of four consecutive quarters were identified. To ensure that only incident cases were included in the analysis, AF diagnoses were considered as initial diagnoses if no AF was diagnosed in at least five consecutive quarters before. Because neither the AVD nor the VDA data provide information about whether a patient is continuously insured in an SHI (vs. private health insurance) or a permanent resident in Germany, the sole absence was not sufficient to exclude a previous AF diagnosis. Thus, only patients with at least one patient-physician contact during the five quarters before the initial AF diagnosis were included. Patients, who fulfilled the diagnosis-related inclusion criteria, remained in the study population if they had received at least one prescription of the oral anticoagulants (OACs) VKA or DOAC after their initial AF diagnosis. To ensure that the OAC prescription was related to the AF diagnosis, prescriptions were only considered if no OAC was prescribed in the two consecutive quarters before the first coded diagnosis. From the time of the initial OAC prescription, the patients were observed until an event of interest was diagnosed or the follow-up period ended. Within the final VKA population about 99.4% were treated with phenprocoumon and 0.6% with warfarin.

## Endpoints and Follow-up

In total, four primary endpoints were defined based on verified outpatient diagnoses (ICD-10-GM, Table S2). To prevent that permanent diagnoses related to past events were considered as first event, diagnoses were only considered if there was no identical diagnosis in the two previous quarters.

Patients who did not experience any primary endpoint during the follow-up period were censored in case of a treatment discontinuation, death or end of the study period. Treatment discontinuation was defined by at last two consecutive quarters of no OAC prescription. Because the data provided no information about mortality, patients were considered dead if they did not get any diagnosis in four consecutive quarters during the follow-up or post-follow-up period (Figure S1, Example 4 and 5). In this case, the time of death was defined by the last quarter any diagnosis was recorded. Patients who did not experience any primary endpoint but died during the follow-up period were censored in case of a treatment discontinuation (Figure S1, Example 3 and 6) or end of the study period (Figure S1, Example 2). For patients who did not experience any endpoint, did not die and continued their treatment, the follow-up ended at the 4^th^ quarter 2016.

Patients, who fulfilled the criterion for death often fulfilled the criterion for treatment discontinuation as well. While a causal relation between death and therapy discontinuation seems plausible at first, a high number of deaths preceded by one quarter of therapy discontinuation rather indicate a particular characteristic of the underlying data. The absence of prescriptions one quarter before death might be related to the forthcoming death rather than a break in therapy. Therefore patients were classed as dead when therapy discontinuation started only one quarter before the criterion for death were met (Figure S1, Example 5).

The chosen criterion for death actually show that when a patient was no longer tracked in the VDA. In theory, this may have various other reasons than death, such as hospitalization, admission to a nursing or rehabilitation home, withdraw from the statutory health insurance or moving to another state or country. In the present patient group with a mean age of 75 years, some of these reasons, like withdrawal and moving, are less likely than others. After hospitalization and rehabilitation, the majority of patients should reappear in the outpatient data within one year. Therefore, after four consecutive quarters without any diagnosis it is very likely but not certain that a patient is dead. For these reasons taken together, the endpoint dead must be interpreted with caution.


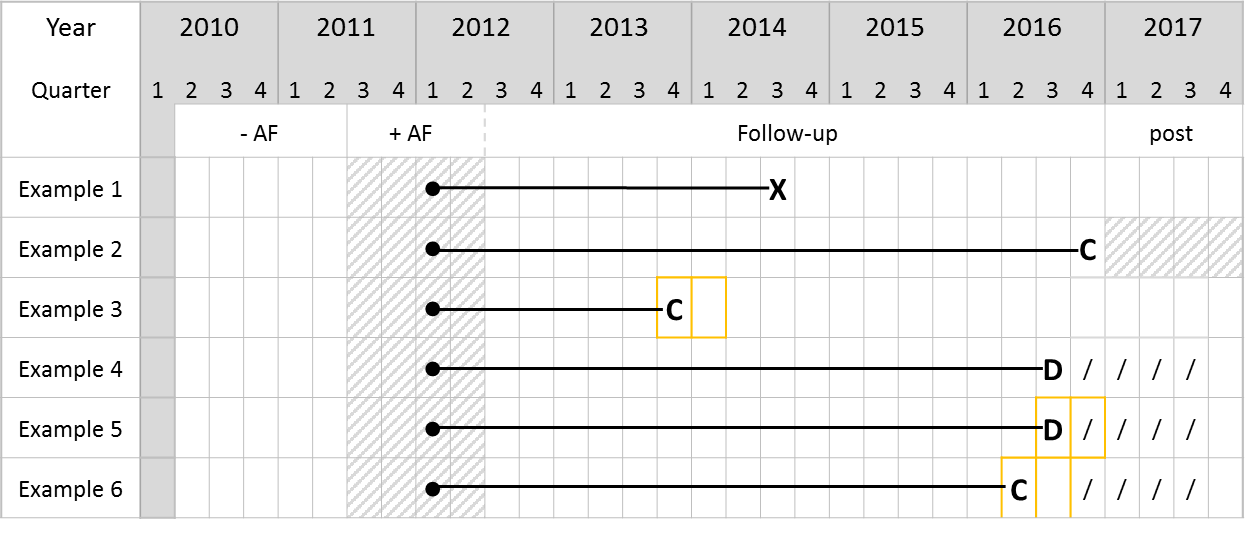


**Figure S1 Follow-up Cohort1** The prerequisites for inclusion were as follows: At least 5 consecutive quarters pre-follow-up period without diagnosis of atrial fibrillation (-AF), followed by at least 2 AF diagnoses within 4 consecutive quarters and an OAC prescription between the 1st AF diagnosis and the 3rd quarter 2016. Follow-up started with 1st OAC prescription (•) until an event of interest (Example 1) or the end of follow-up period. Example 2: Patients with any diagnosis in 2017 where censored (C). Example 3: Two consecutive quarters without any OAC prescription were defined as treatment discontinuation (orange lines) and corresponding patients were censored accordingly. Example 4: Patients without any diagnosis (/) within 4 consecutive quarters at any time during follow-up or post-follow-up period (post) were classed as dead (D). Patients were classed as dead when the criterion of death was accompanied by the criterion of treatment discontinuation (example 5), but were censored when treatment discontinuation occurred before criterion for death were met (example 6).

| Year | | | | 2010 | | | | 2011 | | | | 2012 | | | | 2013 | | | | 2014 | | | | 2015 | | | | 2016 | | | | 2017 | | | |
| --- | --- | --- | --- | --- | --- | --- | --- | --- | --- | --- | --- | --- | --- | --- | --- | --- | --- | --- | --- | --- | --- | --- | --- | --- | --- | --- | --- | --- | --- | --- | --- | --- | --- | --- | --- |
| Quarter | | | | 1 | 2 | 3 | 4 | 1 | 2 | 3 | 4 | 1 | 2 | 3 | 4 | 1 | 2 | 3 | 4 | 1 | 2 | 3 | 4 | 1 | 2 | 3 | 4 | 1 | 2 | 3 | 4 | 1 | 2 | 3 | 4 |
|  |  |  |  |  | -AF | | | | | +AF | | | | Follow-up | | | | | | | | | | | | | | | | | | post | | | |
|  |  |  | 1 |  |  |  |  |  |  |  |  |  |  |  |  |  |  |  |  |  |  |  |  |  |  |  |  |  |  |  |  |  |  |  |  |
|  |  |  | 2 |  |  |  |  |  |  |  |  |  |  |  |  |  |  |  |  |  |  |  |  |  |  |  |  |  |  |  |  |  |  |  |  |
|  |  |  | 3 |  |  |  |  |  |  |  |  |  |  |  |  |  |  |  |  |  |  |  |  |  |  |  |  |  |  |  |  |  |  |  |  |
|  |  |  | 4 |  |  |  |  |  |  |  |  |  |  |  |  |  |  |  |  |  |  |  |  |  |  |  |  |  |  |  |  |  |  |  |  |
|  |  |  | 5 |  |  |  |  |  |  |  |  |  |  |  |  |  |  |  |  |  |  |  |  |  |  |  |  |  |  |  |  |  |  |  |  |
|  |  |  | 6 |  |  |  |  |  |  |  |  |  |  |  |  |  |  |  |  |  |  |  |  |  |  |  |  |  |  |  |  |  |  |  |  |
|  |  |  | 7 |  |  |  |  |  |  |  |  |  |  |  |  |  |  |  |  |  |  |  |  |  |  |  |  |  |  |  |  |  |  |  |  |
|  |  |  | 8 |  |  |  |  |  |  |  |  |  |  |  |  |  |  |  |  |  |  |  |  |  |  |  |  |  |  |  |  |  |  |  |  |
| Cohorts | | | 9 |  |  |  |  |  |  |  |  |  |  |  |  |  |  |  |  |  |  |  |  |  |  |  |  |  |  |  |  |  |  |  |  |
|  |  |  | 10 |  |  |  |  |  |  |  |  |  |  |  |  |  |  |  |  |  |  |  |  |  |  |  |  |  |  |  |  |  |  |  |  |
|  |  |  | 11 |  |  |  |  |  |  |  |  |  |  |  |  |  |  |  |  |  |  |  |  |  |  |  |  |  |  |  |  |  |  |  |  |
|  |  |  | 12 |  |  |  |  |  |  |  |  |  |  |  |  |  |  |  |  |  |  |  |  |  |  |  |  |  |  |  |  |  |  |  |  |
|  |  |  | 13 |  |  |  |  |  |  |  |  |  |  |  |  |  |  |  |  |  |  |  |  |  |  |  |  |  |  |  |  |  |  |  |  |
|  |  |  | 14 |  |  |  |  |  |  |  |  |  |  |  |  |  |  |  |  |  |  |  |  |  |  |  |  |  |  |  |  |  |  |  |  |
|  |  |  | 15 |  |  |  |  |  |  |  |  |  |  |  |  |  |  |  |  |  |  |  |  |  |  |  |  |  |  |  |  |  |  |  |  |
|  |  |  | 16 |  |  |  |  |  |  |  |  |  |  |  |  |  |  |  |  |  |  |  |  |  |  |  |  |  |  |  |  |  |  |  |  |
|  |  |  | 17 |  |  |  |  |  |  |  |  |  |  |  |  |  |  |  |  |  |  |  |  |  |  |  |  |  |  |  |  |  |  |  |  |
|  |  |  | 18 |  |  |  |  |  |  |  |  |  |  |  |  |  |  |  |  |  |  |  |  |  |  |  |  |  |  |  |  |  |  |  |  |
|  |  |  | 19 |  |  |  |  |  |  |  |  |  |  |  |  |  |  |  |  |  |  |  |  |  |  |  |  |  |  |  |  |  |  |  |  |

Figure S2 Cohorts overview

According to the inclusion criteria, a total of 19 cohorts (Figure S2) with pre-follow-up onset in 19 different quarters from the 2nd quarter 2010 until the 4th quarter 2014 could be included in the analysis. The maximal duration of follow-up was 21 quarters (event in quarter 22 – first prescription in quarter 1), the minimum was 1 quarter (event in quarter 22 – first prescription in quarter 21).

The survival time was calculated on quarters because the data did not include any information on whether a diagnosis was made at the end or in the beginning of a quarter. In case the patient experienced an event only one quarter after the first OAC prescription, his/her follow up had a length of one quarter, as shown in Table S1, Example 1 and 2. This means, that for instance, a patient may have had the 1^st^ OAC prescription in the first month and an event after 6 months (Patient 1), or the 1^st^ OAC prescription in the 3^rd^ month and an event after 4 months (Patient 2). In these extreme examples, the follow-up time calculated by months would differ by 4 months but would be the same for both patients when calculated by quarters. However, prescriptions of VKAs should not be generally expected to happen more often in the beginning of a quarter than first prescriptions of DOACs. Likewise, events should not occur more often in the beginning or in the end of a quarter depended on VKA or DOAC treatment. Therefore, the calculation by quarters is not very exact in absolute numbers, but should not bias the comparison between VKAs and DOACs.

Table S1 Quarterly vs. Monthly Follow-up calculation

|  |  | | |  | | | **Follow-up calculation** | |
| --- | --- | --- | --- | --- | --- | --- | --- | --- |
| **Quarter** | **1** | | | **2** | | | Quarters |  |
| **Month** | **1** | **2** | **3** | **4** | **5** | **6** |  | Months |
| Patient 1 | 1^st^ OAC |  |  |  |  | Event | 2-1 = 1 | 6-1 = 5 |
| Patinet 2 |  |  | 1^st^ OAC | Event |  |  | 2-1 = 1 | 4-3 = 1 |

## Treatment classification

Table S2 Treatment classification VKA: Vitamin-K-Antagonist, DOAC: Direct oral anticoagulant, ATC: anatomical therapeutic chemical classification code, Doses: avaliable doses

| **Treatment label** | **Treatment** | **ATC-Code** | **Doses (mg)** |
| --- | --- | --- | --- |
| VKA | Phenprocoumon | B01AA04 | 1.5, 3 |
|  | Warfarin | B01AA03 | 5 |
| DOAC | Dabigatran | B01AE07 | 75, 110, 150 |
|  | Rivaroxaban | B01AF01 | 2.5, 10, 15, 20 |
|  | Apixaban | B01AF02 | 2.5, 5 |
|  | Edoxaban | B01AF03 | 15, 30, 60 |

## Measurements

Diagnoses were identified by the International Statistical Classification of Diseases and Related Health Problems, 10th revision, German Modification (ICD-10-GM).

Medications were identified by the anatomical therapeutic chemical classification codes (ATC-codes).

### Events of Interest

Table S3 Diagnosis codes for the identification of events of interest

| **Events of Interest** | |  | **ICD-10-GM Codes** |
| --- | --- | --- | --- |
| Ischemic Stroke | |  | I63-164 |
| Transient Ischemic Attack (TIA) | |  | G45.- |
| Arterial Embolism | |  | I74.- |
| Bleeding | Extracerebral | Acute bleeding anemia | D62.- |
|  |  | Eye | H11.3-, H21.0-, H31.3-, H35.6-, H43.1-, H45.0- |
|  |  | Ear | H92.2- |
|  |  | Genital tract | N92.0-, N92.1-, N93.8-, N93.9-, N95.0- |
|  |  | Hemopericardium | I23.0-, I31.2-, |
|  |  | Hematothorax | J94.2-, |
|  |  | Hemarthrosis | M25.0- |
|  |  | Respiratory system | R04.- |
|  |  | Skin | R23.3- |
|  | Gastrointestinal | Esophagus | I85.0-, K22.6-, K22.8- |
|  |  | Gastritis, Angiodysplasia & Diverticulosis | K29.0-, K31.82-, K55.22-, K57.01-, K57.03-, K57.11-, K57.13-, K57.83-, K57.91- K57.93-, K62.5- |
|  |  | Hematuria | N02.-, R31.- |
|  |  | Other GI diseases | K66.1-, K92.0-, K92.1-, K92.2- |
|  |  | Ulcus ventriculi,  duodeni &  pepticum jejuni | K25.2-, K25.4-, K25.6-, K26.0-, K26.2-, K26.4-, K26.6-, K27.0-, K27.2-, K27.4-, K27.6-, K28.0-, K28.2-, K28.4-, K28.6- |
|  | Intracerebral |  | I60 - I62 |

To identify events of interest, endpoint related ICD-10 codes were defined based on codes used in previous studies [16, 18, 21] and adapted after consultation with institute clinicians.

### CHA_2_DS_2_-VASc Score

Table S4 Diagnosis codes used for the calculation of the individual stroke risk

| **CHA_2_DS_2_-VASc categories** | **points** | **ICD-10 codes** |
| --- | --- | --- |
| Congestive heart failure | 1 | I50.-, I11.0-, I13.0-, I13.2-, I25.5-, I25.9-, I42.-, I43.- |
| Hypertension | 1 | I10.-, I11.-, I12.-, I13.-, I15.- |
| Age >= 75 years | 2 | - |
| Diabetes mellitus | 1 | E10.-, E11.-, E12.-, E13.-, E14.- |
| Stroke/TIA/thromboembolism | 2 | I61.-, I63.-, I64.-, I69.1-, I69.3-, I69.4-, G45.-, I74.-, I80.-, I81.-, I82.-, I24.0-, I26.-, I67.6-, K55.0-, N28.0-, H34.- |
| Vascular disease | 1 | I21.-, I22.-, I23.-, I25.0-, I25.1-, I25.2-, I25.8-, I73.9-, I70.- |
| Age 65-74 years | 1 | - |
| Female sex | 1 | - |

To calculate an individual CHA_2_DS_2_-VASc score, the age, sex and diagnosed ICD-codes during the year preceding the first OAC prescription were analyzed. In case a patient was diagnosed with at least one ICD-code listed in one of the CHA_2_DS_2_-VASc categories he/she scored one point. For age ≥75 years, or a history of stroke, TIA or thromboembolism the patient scored two points. In case the same patient was diagnosed with multiple codes listed in one category, he/she still scored one point only in the overall assessment. Finally, all points were summed up to one score for each patient, ranging from 0 to 9. ICD-10-codes were based on previous studies [16, 18] and were adapted after consultation with institute clinicians.

### Charlson Comorbidity Score

Table S5 Diagnosis codes used for the calculation of the individual comorbidity

| **Comorbidities** | **Weights** | **ICD-10 codes** |
| --- | --- | --- |
| AIDS/HIV | 6 | B20-B22, B24.- |
| Any malignancy, including lymphoma and leukemia, except malignant neoplasm of skin | 2 | C00-C76, C81-C85, C88-C97 |
| Cerebrovascular disease | 1 | G45-G46, H34.-, I60-I69 |
| Chronic pulmonary disease | 1 | I27.8-, I27.9-, J40-J47, J60-J67, J68.4-, J70.1-, J70.3-, |
| Congestive heart failure | 1 | I09.9-, I11.0-, I13.0-, I13.2-, I25.5-, I42.0-, I42.5-I42.9, I43., I50.-, P29.0 |
| Dementia | 1 | F00-F03, F05.1-, G30.-, G31.1- |
| Diabetes with chronic complication | 2 | E10.2-E10.5, E10.7-, E11.2-E11.5, E11.7-, E12.2-E12.5, E12.7-, E13.2-E13.5, E13.7-, E14.2-E14.5, E14.7- |
| Diabetes without chronic complication | 1 | E10.0-, E10.1-, E10.6-, E10.8-, E10.9-, E11.0-, E11.1-, E11.6-, E11.8-, E11.9-, E12.0-, E12.1-, E12.6-, E12.8-, E12.9-, E13.0-, E13.1-, E13.6-, E13.8-, E13.9-, E14.0-, E14.1-, E14.6-, E14.8-, E14.9- |
| Hemiplegia or paraplegia | 2 | G04.1-, G11.4-, G80.1-, G80.2-, G81-G82, G83.0-G83.4, G83.9 |
| Metastatic solid tumor | 6 | C77-C80 |
| Mild liver disease | 1 | B18.-, K70.0-K70.3, K70.9-, K71.3-K71.5, K73.-, K74.-, K76.0-, K76.2-K76.4, K76.8-, K76.9-, Z94.4- |
| Moderate or severe liver disease | 3 | I85.0-, I85.9-, I86.4-, I98.2-, K70.4- , K71.1-, K72.1, K72.9-, K76.5-, K76.79 |
| Myocardial infarction | 1 | I21-I22, I25.2- |
| Peptic ulcer disease | 1 | K25-K28 |
| Peripheral vascular disease | 1 | I70-I71, I73.1-, I73.8-, I73.9-, I77.1-, I79.0-, I79.1-, K55.1-, K55.8-, K55.9-, Z95.9- |
| Renal disease | 2 | I12.0-, I13.1-, N03.2-N03.7, N05.2-N05.7, N18-N19, N25.0-, Z49.-, Z94.0-, Z99.2- |
| Rheumatic disease | 1 | M05-M06, M32-M34, M31.5-, M35.1-, M35.3-, M36.0- |

To assess individual comorbidities, the Charlson comorbidity score [23] was calculated for each patient based on the diagnosed ICD-codes during the year preceding the first OAC prescription. The ICD-codes were based on an algorithm developed by Quan et al. [24] and modified according to the ICD-10-German modification after Stausberg et al. [26] and Hagn [25]. In case a patient was diagnosed with at least one ICD-code listed in one of the comorbidity-categories he/she scored one point. In case the same patient was diagnosed with multiple codes listed in one category, he/she still scored one point only in the overall assessment. Finally, the points were weighted according to the comorbidity-category as stated in Table S4. The maximum achievable, summed up score for a patient was 29.”

### Comorbidities: Medication

Table S6 ATC-Codes used for the evaluation of risks and comorbidities Including medications directly altering stroke, arterial embolism or bleeding risk or indicating diseases with altered risk of stroke, arterial embolism or bleeding

| **Drug class** | **ATC-Code** |
| --- | --- |
| Antiarrhythmic agents | C08*, C01B*, C07AB*, C07AG* |
| Antihypertensive drugs | C02*, C06A*, C09* |
| Antiplatelet drugs | B01AC* |
| Corticosteroids (systemic use) | H02* |
| Fondaparinux | B01AX05 |
| Heparins | B01AB* |
| Insulin | A10A* |
| Lipid-lowering agents | C10* |
| NSAIDs | M01A*, N02BA01, N02BA51, N02BA71, R05XA02* |
| Oral anti-diabetic drugs | A10B*, A10X* |
| Peptic Ulcer/Reflux disease | A02B* |
| SSRIs | N06AB*, N06AX* |

### Comorbidities: Diagnoses

Table S7 ICD-10-Codes used for the evaluation of comorbidities Including diagnoses related to altered risk of stroke, arterial embolism, bleeding or mortality

| **Comorbidities related to altered risk of stroke, arterial embolism, bleeding or mortality** | |
| --- | --- |
| **Disease class** | **ICD-10-GM Codes** |
| Alcohol abuse/addiction | E24.4-, F10.-, G31.2-, G62.2-, G72.1-, I42.6-, K29.2-, K70.-, K86.0-, T51.-, Z50.2- |
| Bleeding extracerebral | D62.-, H11.3-, H21.0-, H31.3-, H35.6-, H43.1-, H45.0-, H92.2-, I23.0-, I31.2-, J94.2-, M25.0-, N02.-, N92.0-, N92.1-, N93.8-, N93.9-, N95.0-, R04.-, R23.3- |
| Bleeding Gastrointestinal system | I85.0-, K22.6-, K22.8-, K25.2-, K25.4-, K25.6-, K26.0-, K26.2-, K26.4-, K26.6-, K27.0-, K27.2-, K27.4-, K27.6-, K28.0-, K28.2-, K28.4-, K28.6-, K29.0-, K31.82-, K55.22-, K57.01-, K57.03-, K57.11-, K57.13-, K57.83-, K57.91-, K57.93-, K62.5-, K66.1-, K92.0-, K92.1-, K92.2-, R31.-, R58.- |
| Bleeding intracerebral | I60 - I62 |
| Cancer | C00-C97 |
| Coagulopathy | D66-D67, D88.0-, D68.1-, D68.2-, D68.4-, D68.5-, D69.1-, D69.3-, D69.4-, D69.5-, D69.6- |
| Congestive Heart Failure | I11.0-, I13.0-, I13.2-, I42.-, I50.- |
| COPD | J44.- |
| Coronary Heart Disease | I20-I25 |
| Diabetes | E10 -E14 |
| Diverticulitis | K57.- |
| Embolism systemic | I74.- |
| Embolism venous | I26.-, I80-I82 |
| Esophageal varices | I85.- |
| Hypertension | I10-I15 |
| Ischemic Stroke | I63-164, G45.- |
| Liver disease | B15.0-, B16.0-, B16.2-, B19.0-, K70.4-, K72.-, K76.- |
| Nicotin use/dependence | F17.-, Z72.0- |
| Renal disease | I12 - I13, N00 - N05, N07 - N12, N14.-, N17.-, N18.4 - N18.6, N19.-, P96.0-, Z49.1, Z99.2- |
| Upper gastrointestinal system | K20 - K31 |
| Vascular disease | I25.4-, I28.1-, I65.-, I66.-, I67.0-, I67.1-, I70-I72, I79.0- |
| Vascular dementia | F01.- |
| Venous malformation | Q20 - Q28 |

### Sensitivity analysis I: Definition of alternative diagnoses

Table S8 Diagnoses of diseases for which DOAC are also indicated

| **Disease class** | **ICD-10-GM Codes** |
| --- | --- |
| Pulmonary embolism | I26.- |
| Thrombosis, phlebitis and thrombophlebitis | I80.1-I80.9 |
| Presence of a hip joint prosthesis | Z96 |

### Sensitivity analysis II: Definition of approved dosages

Table S9 Available doses of DOAC Approved or not approved for stroke prevention in AF

| **DOAC** | **ATC-Code** | **Dosages** | |
| --- | --- | --- | --- |
|  |  | Approved | Not approved |
| Dabigatran | B01AE07 | 110mg | 75mg |
|  |  | 150mg |  |
| Rivaroxaban | B01AF01 | 15mg | 2.5mg |
|  |  | 20mg | 10mg |
| Apixaban | B01AF02 | 2.5mg |  |
|  |  | 5mg |  |
| Edoxaban | B01AF03 | 15mg |  |
|  |  | 30mg |  |
|  |  | 60mg |  |

## R-Packages

The following R-Packages (version number) were used for the analysis:

- cobalt (3.7.0) [31]
- dplyr (0.8.3) [32]
- ggplot2 (3.2.1) [33]
- gridExtra (2.3) [34]
- MatchIt (3.0.2) [35]
- ROracle (1.3-1.) [35]
- survival (2.44-1.1) [36]

# Results

## Joint DOAC Analysis

### Patient Characteristics before matching

#### Stroke Population

Table S10

| **Patient characteristics stroke population** | | **VKA(n=405437)** | **DOAC(n=430940)** |
| --- | --- | --- | --- |
| Age | Mean(± SD) | 75.71(±8.82) | 75.48(±9.98) |
|  | Median | 77 | 77 |
| Age distribution (%) | 18-36 | 0.09 | 0.2 |
|  | 37-54 | 2.17 | 3.41 |
|  | 55-72 | 27.65 | 28.31 |
|  | 73+ | 70.09 | 68.08 |
| Female Sex (%) |  | 51.1 | 54.66 |
| CHA_2_DS_2_-VASc Score | Mean(± SD) | 4.48(±1.71) | 4.28(±1.77) |
|  | Median | 4 | 4 |
| CHA_2_DS_2_-VASc Score distribution (%) | 0-1 | 3.71 | 5.93 |
|  | 2-3 | 24.73 | 26.9 |
|  | 4-5 | 44.59 | 42.98 |
|  | 6-7 | 22.77 | 20.48 |
|  | 8-9 | 4.19 | 3.71 |
| Charlson Comorbidity Index | Mean(± SD) | 2.98(±2.66) | 2.74(±2.6) |
|  | Median | 2 | 2 |
| Charlson Comorbidity Index distribution (%) | 0-4 | 75.97 | 78.92 |
|  | 5-9 | 21.58 | 18.89 |
|  | 10-15 | 2.35 | 2.11 |
|  | 15+ | 0.17 | 0.13 |
| Number of distinct prescriptions (ATCs) | Mean(± SD) | 12.68(±5.77) | 12.42(±5.77) |
|  | Median | 12 | 11 |
| Number of prescriptions (ATCs) | Mean(± SD) | 36.03(±21.54) | 34.34(±21.1) |
|  | Median | 31 | 30 |
| Number of diagnoses (ICD-Codes) | Mean(± SD) | 28.49(±15.51) | 27.88(±15.53) |
|  | Median | 26 | 25 |
| Prescribed Medicines (%) | Antiarrhythmic agents | 90.65 | 90.33 |
|  | Antihypertensive drugs | 81.38 | 79.01 |
|  | Antiplatelet drugs | 24.98 | 26.09 |
|  | Corticosteroids (systemic use) | 14.76 | 15.7 |
|  | Fondaparinux | 0.8 | 0.58 |
|  | Heparins | 36.44 | 18.91 |
|  | Insulin | 11.25 | 10.09 |
|  | Lipid-lowering agents | 48.47 | 43.99 |
|  | NSAIDs | 39.76 | 42.41 |
|  | Oral anti-diabetic drugs | 20.14 | 19.91 |
|  | Peptic Ulcer/Reflux disease | 50.04 | 51.79 |
|  | SSRIs | 5.21 | 6.12 |
| Comorbidities (%) | Alcohol abuse/addiction | 2.28 | 2.62 |
|  | Bleeding extracerebral | 5.81 | 4.71 |
|  | Bleeding gastrointestinal | 6.04 | 5.57 |
|  | Bleeding intracerebral | 0.42 | 0.55 |
|  | Cancer | 19.96 | 20.03 |
|  | Coagulopathy | 1.94 | 1.46 |
|  | Congestive Heart Failure | 29.82 | 24.54 |
|  | COPD | 16.2 | 15.46 |
|  | Coronary Heart Disease | 41.73 | 35.35 |
|  | Diabetes | 39.1 | 36.63 |
|  | Diverticulitis | 10.67 | 11.11 |
|  | Embolism systemic | 0.88 | 0.6 |
|  | Embolism venous | 5.48 | 4.32 |
|  | Esophageal varices | 0.21 | 0.22 |
|  | Hypertension | 88.4 | 86.89 |
|  | Ischemic Stroke | 8.69 | 8.21 |
|  | Liver disease | 13.61 | 13.07 |
|  | Nicotin use/dependence | 4.44 | 4.66 |
|  | Renal disease | 15.91 | 11.48 |
|  | Upper gastrointestinal system | 25.99 | 27.17 |
|  | Vascular disease | 22.43 | 20.68 |
|  | Vascular dementia | 1.73 | 2.36 |
|  | Venous malformation | 1.99 | 1.43 |

#### TIA Population

Table S11

| **Patient characteristics TIA population** | | VKA (n=405012) | DOAC (n=431574) |
| --- | --- | --- | --- |
| Age | Mean(± SD) | 75.71(±8.82) | 75.48(±9.98) |
|  | Median | 77 | 77 |
| Age distribution (%) | 18-36 | 0.09 | 0.2 |
|  | 37-54 | 2.17 | 3.41 |
|  | 55-72 | 27.65 | 28.3 |
|  | 73+ | 70.09 | 68.09 |
| Female Sex (%) |  | 51.1 | 54.66 |
| CHA_2_DS_2_-VASc Score | Mean(± SD) | 4.48(±1.71) | 4.28(±1.77) |
|  | Median | 4 | 4 |
| CHA_2_DS_2_-VASc Score distribution (%) | 0-1 | 3.71 | 5.92 |
|  | 2-3 | 24.74 | 26.89 |
|  | 4-5 | 44.61 | 42.97 |
|  | 6-7 | 22.76 | 20.49 |
|  | 8-9 | 4.18 | 3.73 |
| Charlson Comorbidity Index | Mean(± SD) | 2.98(±2.66) | 2.74(±2.6) |
|  | Median | 2 | 2 |
| Charlson Comorbidity Index distribution (%) | 0-4 | 75.99 | 78.9 |
|  | 5-9 | 21.57 | 18.91 |
|  | 10-15 | 2.35 | 2.12 |
|  | 15+ | 0.17 | 0.13 |
| Number of distinct prescriptions (ATCs) | Mean(± SD) | 12.68(±5.76) | 12.42(±5.77) |
|  | Median | 12 | 11 |
| Number of prescriptions (ATCs) | Mean(± SD) | 36.02(±21.53) | 34.35(±21.11) |
|  | Median | 31 | 30 |
| Number of diagnoses (ICD-Codes) | Mean(± SD) | 28.49(±15.51) | 27.89(±15.53) |
|  | Median | 26 | 25 |
| Prescribed Medicines (%) | Antiarrhythmic agents | 90.65 | 90.33 |
|  | Antihypertensive drugs | 81.38 | 79.01 |
|  | Antiplatelet drugs | 24.96 | 26.12 |
|  | Corticosteroids (systemic use) | 14.77 | 15.69 |
|  | Fondaparinux | 0.8 | 0.58 |
|  | Heparins | 36.44 | 18.93 |
|  | Insulin | 11.24 | 10.09 |
|  | Lipid-lowering agents | 48.46 | 44.02 |
|  | NSAIDs | 39.76 | 42.42 |
|  | Oral anti-diabetic drugs | 20.13 | 19.92 |
|  | Peptic Ulcer/Reflux disease drugs | 50.03 | 51.79 |
|  | SSRIs | 5.2 | 6.13 |
| Comorbidities (%) | Alcohol abuse/addiction | 2.28 | 2.62 |
|  | Bleeding Extracerebral | 5.81 | 4.71 |
|  | Bleeding GI | 6.04 | 5.57 |
|  | Bleeding Intracerebral | 0.42 | 0.55 |
|  | Cancer | 19.96 | 20.03 |
|  | Coagulopathy | 1.94 | 1.46 |
|  | Congestive Heart Failure | 29.82 | 24.55 |
|  | COPD | 16.2 | 15.46 |
|  | Coronary Heart Disease | 41.73 | 35.36 |
|  | Diabetes | 39.09 | 36.65 |
|  | Diverticulitis | 10.68 | 11.1 |
|  | Embolism systemic | 0.88 | 0.61 |
|  | Embolism venous | 5.48 | 4.32 |
|  | Esophageal varices | 0.21 | 0.22 |
|  | Hypertension | 88.4 | 86.89 |
|  | Ischemic Stroke | 8.65 | 8.24 |
|  | Liver disease | 13.61 | 13.07 |
|  | Nicotin use/dependence | 4.44 | 4.66 |
|  | Renal disease | 15.92 | 11.48 |
|  | Upper GI | 26 | 27.17 |
|  | Vascular disease | 22.43 | 20.69 |
|  | Vascular dementia | 1.72 | 2.37 |
|  | Venous malformation | 1.99 | 1.42 |

#### Embolism Population

Table S12

| **Patient characteristics embolism population** | | **VKA(n=404823)** | **DOAC(n=431816)** |
| --- | --- | --- | --- |
| Age | Mean(± SD) | 75.71(±8.82) | 75.48(±9.98) |
|  | Median | 77 | 77 |
| Age distribution (%) | 18-36 | 0.09 | 0.2 |
|  | 37-54 | 2.17 | 3.41 |
|  | 55-72 | 27.65 | 28.3 |
|  | 73+ | 70.08 | 68.09 |
| Female Sex (%) |  | 51.1 | 54.66 |
| CHA_2_DS_2_-VASc Score | Mean(± SD) | 4.48(±1.71) | 4.28(±1.77) |
|  | Median | 4 | 4 |
| CHA_2_DS_2_-VASc Score distribution (%) | 0-1 | 3.71 | 5.92 |
|  | 2-3 | 24.75 | 26.88 |
|  | 4-5 | 44.6 | 42.98 |
|  | 6-7 | 22.76 | 20.49 |
|  | 8-9 | 4.18 | 3.73 |
| Charlson Comorbidity Index | Mean(± SD) | 2.97(±2.66) | 2.74(±2.6) |
|  | Median | 2 | 2 |
| Charlson Comorbidity Index distribution (%) | 0-4 | 75.98 | 78.9 |
|  | 5-9 | 21.57 | 18.91 |
|  | 10-15 | 2.35 | 2.11 |
|  | 15+ | 0.17 | 0.13 |
| Number of distinct prescriptions (ATCs) | Mean(± SD) | 12.68(±5.76) | 12.42(±5.77) |
|  | Median | 12 | 11 |
| Number of prescriptions (ATCs) | Mean(± SD) | 36.02(±21.53) | 34.35(±21.11) |
|  | Median | 31 | 30 |
| Number of diagnoses (ICD-Codes) | Mean(± SD) | 28.49(±15.51) | 27.89(±15.53) |
|  | Median | 26 | 25 |
| Prescribed Medicines (%) | Antiarrhythmic agents | 90.66 | 90.33 |
|  | Antihypertensive drugs | 81.37 | 79.02 |
|  | Antiplatelet drugs | 24.95 | 26.12 |
|  | Corticosteroids | 14.77 | 15.69 |
|  | Fondaparinux | 0.79 | 0.59 |
|  | Heparins | 36.43 | 18.95 |
|  | Insulin | 11.24 | 10.09 |
|  | Lipid-lowering agents | 48.45 | 44.03 |
|  | NSAIDs | 39.75 | 42.42 |
|  | Oral anti-diabetic drugs | 20.12 | 19.93 |
|  | Peptic Ulcer/Reflux disease | 50.04 | 51.79 |
|  | SSRIs | 5.2 | 6.13 |
| Comorbidities (%) | Alcohol abuse/addiction | 2.28 | 2.62 |
|  | Bleeding extracerebral | 5.81 | 4.71 |
|  | Bleeding gastrointestinal | 6.04 | 5.57 |
|  | Bleeding intracerebral | 0.42 | 0.55 |
|  | Cancer | 19.96 | 20.03 |
|  | Coagulopathy | 1.94 | 1.46 |
|  | Congestive Heart Failure | 29.82 | 24.55 |
|  | COPD | 16.2 | 15.46 |
|  | Coronary Heart Disease | 41.73 | 35.37 |
|  | Diabetes | 39.08 | 36.65 |
|  | Diverticulitis | 10.67 | 11.11 |
|  | Embolism systemic | 0.88 | 0.6 |
|  | Embolism venous | 5.48 | 4.32 |
|  | Esophageal varices | 0.21 | 0.22 |
|  | Hypertension | 88.4 | 86.89 |
|  | Ischemic Stroke | 8.63 | 8.26 |
|  | Liver disease | 13.61 | 13.06 |
|  | Nicotin use/dependence | 4.44 | 4.66 |
|  | Renal disease | 15.92 | 11.48 |
|  | Upper gastrointestinal system | 25.99 | 27.17 |
|  | Vascular disease | 22.43 | 20.69 |
|  | Vascular dementia | 1.72 | 2.37 |
|  | Venous malformation | 1.99 | 1.42 |

#### Bleeding Population

Table S13

| **Patient characteristics bleeding population** | | **VKA(n=408402)** | **DOAC(n=427477)** |
| --- | --- | --- | --- |
| Age | Mean(± SD) | 75.7(±8.81) | 75.48(±10) |
|  | Median | 77 | 77 |
| Age distribution (%) | 18-36 | 0.09 | 0.2 |
|  | 37-54 | 2.16 | 3.42 |
|  | 55-72 | 27.67 | 28.29 |
|  | 73+ | 70.07 | 68.09 |
| Female Sex (%) |  | 51.14 | 54.65 |
| CHA_2_DS_2_-VASc Score | Mean(± SD) | 4.48(±1.71) | 4.28(±1.77) |
|  | Median | 4 | 4 |
| CHA_2_DS_2_-VASc Score distribution (%) | 0-1 | 3.71 | 5.95 |
|  | 2-3 | 24.74 | 26.91 |
|  | 4-5 | 44.63 | 42.93 |
|  | 6-7 | 22.75 | 20.49 |
|  | 8-9 | 4.18 | 3.72 |
| Charlson Comorbidity Index | Mean(± SD) | 2.97(±2.66) | 2.74(±2.6) |
|  | Median | 2 | 2 |
| Charlson Comorbidity Index distribution (%) | 0-4 | 76 | 78.91 |
|  | 5-9 | 21.55 | 18.9 |
|  | 10-15 | 2.36 | 2.11 |
|  | 15+ | 0.17 | 0.13 |
| Number of distinct prescriptions (ATCs) | Mean(± SD) | 12.69(±5.77) | 12.41(±5.76) |
|  | Median | 12 | 11 |
| Number of prescriptions (ATCs) | Mean(± SD) | 36.04(±21.54) | 34.32(±21.09) |
|  | Median | 31 | 30 |
| Number of diagnoses (ICD-Codes) | Mean(± SD) | 28.52(±15.52) | 27.84(±15.52) |
|  | Median | 26 | 25 |
| Prescribed Medicines (%) | Antiarrhythmic agents | 90.65 | 90.33 |
|  | Antihypertensive drugs | 81.39 | 78.99 |
|  | Antiplatelet drugs | 24.96 | 26.12 |
|  | Corticosteroids | 14.78 | 15.69 |
|  | Fondaparinux | 0.8 | 0.58 |
|  | Heparins | 36.56 | 18.65 |
|  | Insulin | 11.26 | 10.07 |
|  | Lipid-lowering agents | 48.43 | 44 |
|  | NSAIDs | 39.84 | 42.35 |
|  | Oral anti-diabetic drugs | 20.18 | 19.87 |
|  | Peptic Ulcer/Reflux disease | 50.04 | 51.8 |
|  | SSRIs | 5.21 | 6.13 |
| Comorbidities (%) | Alcohol abuse/addiction | 2.27 | 2.62 |
|  | Bleeding extracerebral | 5.84 | 4.66 |
|  | Bleeding gastrointestinal | 6.06 | 5.54 |
|  | Bleeding intracerebral | 0.42 | 0.55 |
|  | Cancer | 19.97 | 20.02 |
|  | Coagulopathy | 1.93 | 1.46 |
|  | Congestive Heart Failure | 29.79 | 24.53 |
|  | COPD | 16.22 | 15.44 |
|  | Coronary Heart Disease | 41.72 | 35.31 |
|  | Diabetes | 39.13 | 36.59 |
|  | Diverticulitis | 10.69 | 11.09 |
|  | Embolism systemic | 0.88 | 0.6 |
|  | Embolism venous | 5.49 | 4.3 |
|  | Esophageal varices | 0.21 | 0.22 |
|  | Hypertension | 88.41 | 86.86 |
|  | Ischemic Stroke | 8.63 | 8.27 |
|  | Liver disease | 13.63 | 13.04 |
|  | Nicotin use/dependence | 4.44 | 4.66 |
|  | Renal disease | 15.88 | 11.48 |
|  | Upper gastrointestinal system | 26.03 | 27.15 |
|  | Vascular disease | 22.42 | 20.68 |
|  | Vascular dementia | 1.72 | 2.38 |
|  | Venous malformation | 1.99 | 1.42 |

#### Mortality Population

Table S14

| **Patient characteristics mortality population** | | **VKA(n=404695)** | **DOAC(n=431978)** |
| --- | --- | --- | --- |
| Age | Mean(± SD) | 75.71(±8.82) | 75.48(±9.98) |
|  | Median | 77 | 77 |
| Age distribution (%) | 18-36 | 0.09 | 0.2 |
|  | 37-54 | 2.17 | 3.41 |
|  | 55-72 | 27.66 | 28.3 |
|  | 73+ | 70.08 | 68.09 |
| Female Sex (%) |  | 51.1 | 54.66 |
| CHA_2_DS_2_-VASc Score | Mean(± SD) | 4.48(±1.71) | 4.28(±1.77) |
|  | Median | 4 | 4 |
| CHA_2_DS_2_-VASc Score distribution (%) | 0-1 | 3.71 | 5.92 |
|  | 2-3 | 24.75 | 26.88 |
|  | 4-5 | 44.6 | 42.97 |
|  | 6-7 | 22.76 | 20.5 |
|  | 8-9 | 4.17 | 3.73 |
| Charlson Comorbidity Index | Mean(± SD) | 2.97(±2.66) | 2.74(±2.6) |
|  | Median | 2 | 2 |
| Charlson Comorbidity Index distribution (%) | 0-4 | 75.99 | 78.9 |
|  | 5-9 | 21.56 | 18.91 |
|  | 10-15 | 2.35 | 2.12 |
|  | 15+ | 0.17 | 0.13 |
| Number of distinct prescriptions (ATCs) | Mean(± SD) | 12.68(±5.76) | 12.42(±5.77) |
|  | Median | 12 | 11 |
| Number of prescriptions (ATCs) | Mean(± SD) | 36.02(±21.53) | 34.35(±21.11) |
|  | Median | 31 | 30 |
| Number of diagnoses (ICD-Codes) | Mean(± SD) | 28.48(±15.51) | 27.89(±15.54) |
|  | Median | 26 | 25 |
| Prescribed Medicines (%) | Antiarrhythmic agents | 90.66 | 90.33 |
|  | Antihypertensive drugs | 81.38 | 79.02 |
|  | Antiplatelet drugs | 24.95 | 26.12 |
|  | Corticosteroids (systemic use) | 14.77 | 15.69 |
|  | Fondaparinux | 0.8 | 0.59 |
|  | Heparins | 36.42 | 18.96 |
|  | Insulin | 11.24 | 10.09 |
|  | Lipid-lowering agents | 48.45 | 44.03 |
|  | NSAIDs | 39.75 | 42.42 |
|  | Oral anti-diabetic drugs | 20.13 | 19.92 |
|  | Peptic Ulcer/Reflux disease drugs | 50.03 | 51.79 |
|  | SSRIs | 5.2 | 6.13 |
| Comorbidities (%) | Alcohol abuse/addiction | 2.28 | 2.62 |
|  | Bleeding extracerebral | 5.81 | 4.71 |
|  | Bleeding gastrointestinal | 6.04 | 5.57 |
|  | Bleeding intracerebral | 0.42 | 0.55 |
|  | Cancer | 19.96 | 20.03 |
|  | Coagulopathy | 1.94 | 1.46 |
|  | Congestive Heart Failure | 29.82 | 24.55 |
|  | COPD | 16.2 | 15.46 |
|  | Coronary Heart Disease | 41.73 | 35.37 |
|  | Diabetes | 39.08 | 36.65 |
|  | Diverticulitis | 10.67 | 11.11 |
|  | Embolism systemic | 0.88 | 0.61 |
|  | Embolism venous | 5.48 | 4.32 |
|  | Esophageal varices | 0.21 | 0.22 |
|  | Hypertension | 88.4 | 86.89 |
|  | Ischemic Stroke | 8.63 | 8.26 |
|  | Liver disease | 13.61 | 13.06 |
|  | Nicotin use/dependence | 4.44 | 4.66 |
|  | Renal disease | 15.92 | 11.48 |
|  | Upper gastrointestinal system | 25.99 | 27.17 |
|  | Vascular disease | 22.43 | 20.69 |
|  | Vascular dementia | 1.72 | 2.37 |
|  | Venous malformation | 1.99 | 1.43 |

### Patient Characteristics after matching

#### TIA Population

Table S15

| **Patient characteristics TIA population**  **After matching** | | **VKA**  (n=347247) | **DOAC**  (n=347247) | **Smd** | |
| --- | --- | --- | --- | --- | --- |
|  |  |  |  | Before | After |
| Age | Mean(± SD) | 75.77(±8.85) | 75.69(±9.85) | 0.03 | 0.01 |
|  | Median | 77 | 77 |  |  |
| Age distribution (%) | 18-36 | 0.09 | 0.2 |  |  |
|  | 37-54 | 2.16 | 3.21 |  |  |
|  | 55-72 | 27.54 | 27.48 |  |  |
|  | 73+ | 70.21 | 69.11 |  |  |
| Female Sex (%) |  | 51.99 | 52.93 | -0.07 | -0.02 |
| CHA_2_DS_2_-VASc Score | Mean(± SD) | 4.45(±1.7) | 4.39(±1.77) | 0.12 | 0.04 |
|  | Median | 4 | 4 |  |  |
| CHA_2_DS_2_-VASc Score distribution (%) | 0-1 | 3.78 | 5.29 |  |  |
|  | 2-3 | 25.12 | 25.34 |  |  |
|  | 4-5 | 44.81 | 43.19 |  |  |
|  | 6-7 | 22.34 | 21.98 |  |  |
|  | 8-9 | 3.94 | 4.2 |  |  |
| Charlson Comorbidity  Index | Mean(± SD) | 2.92(±2.63) | 2.84(±2.63) | 0.09 | 0.03 |
|  | Median | 2 | 2 |  |  |
| Charlson Comorbidity  Index distribution (%) | 0-4 | 76.8 | 77.67 |  |  |
|  | 5-9 | 20.87 | 20.03 |  |  |
|  | 10-15 | 2.23 | 2.22 |  |  |
|  | 15+ | 0.16 | 0.14 |  |  |
| Number of distinct  prescriptions (ATCs) | Mean(± SD) | 12.5(±5.72) | 12.43(±5.78) | 0.05 | 0.01 |
|  | Median | 12 | 11 |  |  |
| Number of  prescriptions (ATCs) | Mean(± SD) | 35.43(±21.17) | 34.88(±21.45) | 0.08 | 0.03 |
|  | Median | 31 | 30 |  |  |
| Number of diagnoses  (ICD-Codes) | Mean(± SD) | 28.21(±15.41) | 27.97(±15.54) | 0.04 | 0.02 |
|  | Median | 26 | 25 |  |  |
| Prescribed Medicines  (%) | Antiarrhythmic agents | 90.47 | 90.43 | 0.01 | 0.00 |
|  | Antihypertensive drugs | 80.9 | 80.38 | 0.06 | 0.01 |
|  | Antiplatelet drugs | 25.06 | 25.56 | -0.03 | -0.01 |
|  | Corticosteroids (systemic use) | 14.85 | 15.03 | -0.03 | -0.01 |
|  | Fondaparinux | 0.78 | 0.68 | 0.02 | 0.01 |
|  | Heparins | 27.12 | 23.5 | 0.36 | 0.08 |
|  | Insulin | 10.95 | 10.56 | 0.04 | 0.01 |
|  | Lipid-lowering agents | 47.56 | 46.34 | 0.09 | 0.02 |
|  | NSAIDs | 40.02 | 40.88 | -0.05 | -0.02 |
|  | Oral anti-diabetic drugs | 20.1 | 20.16 | 0.01 | 0.00 |
|  | Peptic Ulcer/Reflux disease | 49.92 | 50.26 | -0.04 | -0.01 |
|  | SSRIs | 5.4 | 5.58 | -0.04 | -0.01 |
| Comorbidities (%) | Alcohol abuse/addiction | 2.34 | 2.4 | -0.02 | 0.00 |
|  | Bleeding extracerebral | 5.53 | 5.12 | 0.05 | 0.02 |
|  | Bleeding gastrointestinal | 5.84 | 5.67 | 0.02 | 0.01 |
|  | Bleeding intracerebral | 0.44 | 0.47 | -0.02 | 0.00 |
|  | Cancer | 19.77 | 19.78 | 0.00 | 0.00 |
|  | Coagulopathy | 1.82 | 1.64 | 0.03 | 0.01 |
|  | Congestive Heart Failure | 28.96 | 27.22 | 0.12 | 0.04 |
|  | COPD | 16.07 | 15.77 | 0.02 | 0.01 |
|  | Coronary Heart Disease | 40.58 | 38.68 | 0.13 | 0.04 |
|  | Diabetes | 38.62 | 37.93 | 0.05 | 0.01 |
|  | Diverticulitis | 10.65 | 10.83 | -0.01 | -0.01 |
|  | Embolism systemic | 0.81 | 0.7 | 0.03 | 0.01 |
|  | Embolism venous | 5.16 | 4.74 | 0.05 | 0.02 |
|  | Esophageal varices | 0.22 | 0.21 | 0.00 | 0.00 |
|  | Hypertension | 88.1 | 87.77 | 0.05 | 0.01 |
|  | Ischemic Stroke | 8.62 | 8.42 | 0.01 | 0.01 |
|  | Liver disease | 13.46 | 13.31 | 0.02 | 0.00 |
|  | Nicotin use/dependence | 4.46 | 4.49 | -0.01 | 0.00 |
|  | Renal disease | 14.86 | 13.32 | 0.12 | 0.04 |
|  | Upper gastrointestinal system | 26.01 | 26.28 | -0.03 | -0.01 |
|  | Vascular disease | 22.06 | 21.5 | 0.04 | 0.01 |
|  | Vascular dementia | 1.86 | 1.97 | -0.05 | -0.01 |
|  | Venous malformation | 1.86 | 1.64 | 0.04 | 0.02 |

#### Embolism Population

Table S16

| **Patient characteristics embolism population**  **After matching** | | **VKA**  (n=347297) | **DOAC**  (n=347297) | **Smd** | |
| --- | --- | --- | --- | --- | --- |
|  |  |  |  | Before | After |
| Age | Mean(± SD) | 75.77(±8.85) | 75.69(±9.84) | 0.03 | 0.01 |
|  | Median | 77 | 77 |  |  |
| Age distribution (%) | 18-36 | 0.09 | 0.19 |  |  |
|  | 37-54 | 2.16 | 3.21 |  |  |
|  | 55-72 | 27.53 | 27.47 |  |  |
|  | 73+ | 70.22 | 69.12 |  |  |
| Female Sex (%) |  | 52.04 | 52.96 | -0.07 | -0.02 |
| CHA_2_DS_2_-VASc Score | Mean(± SD) | 4.45(±1.7) | 4.39(±1.77) | 0.12 | 0.04 |
|  | Median | 4 | 4 |  |  |
| CHA_2_DS_2_-VASc Score distribution (%) | 0-1 | 3.77 | 5.31 |  |  |
|  | 2-3 | 25.16 | 25.3 |  |  |
|  | 4-5 | 44.78 | 43.16 |  |  |
|  | 6-7 | 22.37 | 22.04 |  |  |
|  | 8-9 | 3.93 | 4.19 |  |  |
| Charlson Comorbidity  Index | Mean(± SD) | 2.92(±2.63) | 2.84(±2.63) | 0.09 | 0.03 |
|  | Median | 2 | 2 |  |  |
| Charlson Comorbidity  Index distribution (%) | 0-4 | 76.77 | 77.65 |  |  |
|  | 5-9 | 20.89 | 20.03 |  |  |
|  | 10-15 | 2.24 | 2.24 |  |  |
|  | 15+ | 0.16 | 0.14 |  |  |
| Number of distinct  prescriptions (ATCs) | Mean(± SD) | 12.5(±5.73) | 12.43(±5.78) | 0.05 | 0.01 |
|  | Median | 12 | 11 |  |  |
| Number of  prescriptions (ATCs) | Mean(± SD) | 35.43(±21.18) | 34.89(±21.44) | 0.08 | 0.02 |
|  | Median | 31 | 30 |  |  |
| Number of diagnoses  (ICD-Codes) | Mean(± SD) | 28.2(±15.4) | 27.99(±15.55) | 0.04 | 0.01 |
|  | Median | 26 | 25 |  |  |
| Prescribed Medicines  (%) | Antiarrhythmic agents | 90.49 | 90.39 | 0.01 | 0.00 |
|  | Antihypertensive drugs | 80.91 | 80.42 | 0.06 | 0.01 |
|  | Antiplatelet drugs | 25.12 | 25.57 | -0.03 | -0.01 |
|  | Corticosteroids (systemic use) | 14.83 | 15.08 | -0.03 | -0.01 |
|  | Fondaparinux | 0.77 | 0.69 | 0.02 | 0.01 |
|  | Heparins | 27.16 | 23.53 | 0.36 | 0.08 |
|  | Insulin | 10.95 | 10.58 | 0.04 | 0.01 |
|  | Lipid-lowering agents | 47.59 | 46.41 | 0.09 | 0.02 |
|  | NSAIDs | 39.94 | 40.79 | -0.05 | -0.02 |
|  | Oral anti-diabetic drugs | 20.09 | 20.14 | 0.00 | 0.00 |
|  | Peptic Ulcer/Reflux disease | 49.9 | 50.36 | -0.04 | -0.01 |
|  | SSRIs | 5.38 | 5.58 | -0.04 | -0.01 |
| Comorbidities (%) | Alcohol abuse/addiction | 2.33 | 2.42 | -0.02 | -0.01 |
|  | Bleeding extracerebral | 5.51 | 5.13 | 0.05 | 0.02 |
|  | Bleeding gastrointestinal | 5.85 | 5.69 | 0.02 | 0.01 |
|  | Bleeding intracerebral | 0.44 | 0.47 | -0.02 | -0.01 |
|  | Cancer | 19.76 | 19.77 | 0.00 | 0.00 |
|  | Coagulopathy | 1.82 | 1.64 | 0.03 | 0.01 |
|  | Congestive Heart Failure | 28.92 | 27.19 | 0.12 | 0.04 |
|  | COPD | 16.04 | 15.8 | 0.02 | 0.01 |
|  | Coronary Heart Disease | 40.56 | 38.76 | 0.13 | 0.04 |
|  | Diabetes | 38.64 | 37.94 | 0.05 | 0.01 |
|  | Diverticulitis | 10.66 | 10.76 | -0.01 | 0.00 |
|  | Embolism systemic | 0.81 | 0.7 | 0.03 | 0.01 |
|  | Embolism venous | 5.15 | 4.75 | 0.05 | 0.02 |
|  | Esophageal varices | 0.21 | 0.22 | 0.00 | 0.00 |
|  | Hypertension | 88.11 | 87.81 | 0.05 | 0.01 |
|  | Ischemic Stroke | 8.55 | 8.4 | 0.01 | 0.01 |
|  | Liver disease | 13.45 | 13.3 | 0.02 | 0.00 |
|  | Nicotin use/dependence | 4.47 | 4.49 | -0.01 | 0.00 |
|  | Renal disease | 14.85 | 13.32 | 0.12 | 0.04 |
|  | Upper gastrointestinal system | 26.06 | 26.36 | -0.03 | -0.01 |
|  | Vascular disease | 22.02 | 21.49 | 0.04 | 0.01 |
|  | Vascular dementia | 1.86 | 1.98 | -0.05 | -0.01 |
|  | Venous malformation | 1.87 | 1.64 | 0.04 | 0.02 |

#### Bleeding Population

Table S17

| **Patient characteristics bleeding population**  **After matching** | | **VKA**  (n=346343) | **DOAC**  (n=346343) | **Smd** | |
| --- | --- | --- | --- | --- | --- |
|  |  |  |  | Before | After |
| Age | Mean(± SD) | 75.77(±8.85) | 75.69(±9.86) | 0.02 | 0.01 |
|  | Median | 77 | 77 |  |  |
| Age distribution (%) | 18-36 | 0.09 | 0.2 |  |  |
|  | 37-54 | 2.15 | 3.21 |  |  |
|  | 55-72 | 27.53 | 27.46 |  |  |
|  | 73+ | 70.22 | 69.13 |  |  |
| Female Sex (%) |  | 52.05 | 53.03 | -0.07 | -0.02 |
| CHA_2_DS_2_-VASc Score | Mean(± SD) | 4.45(±1.7) | 4.38(±1.77) | 0.12 | 0.04 |
|  | Median | 4 | 4 |  |  |
| CHA_2_DS_2_-VASc Score distribution (%) | 0-1 | 3.76 | 5.31 |  |  |
|  | 2-3 | 25.12 | 25.38 |  |  |
|  | 4-5 | 44.85 | 43.18 |  |  |
|  | 6-7 | 22.31 | 21.98 |  |  |
|  | 8-9 | 3.95 | 4.16 |  |  |
| Charlson Comorbidity  Index | Mean(± SD) | 2.92(±2.63) | 2.83(±2.63) | 0.09 | 0.03 |
|  | Median | 2 | 2 |  |  |
| Charlson Comorbidity  Index distribution (%) | 0-4 | 76.85 | 77.77 |  |  |
|  | 5-9 | 20.84 | 19.93 |  |  |
|  | 10-15 | 2.22 | 2.21 |  |  |
|  | 15+ | 0.16 | 0.14 |  |  |
| Number of distinct  prescriptions (ATCs) | Mean(± SD) | 12.48(±5.72) | 12.42(±5.77) | 0.05 | 0.01 |
|  | Median | 12 | 11 |  |  |
| Number of  prescriptions (ATCs) | Mean(± SD) | 35.39(±21.17) | 34.85(±21.43) | 0.08 | 0.03 |
|  | Median | 31 | 30 |  |  |
| Number of diagnoses  (ICD-Codes) | Mean(± SD) | 28.2(±15.39) | 27.93(±15.52) | 0.04 | 0.02 |
|  | Median | 26 | 25 |  |  |
| Prescribed Medicines  (%) | Antiarrhythmic agents | 90.46 | 90.34 | 0.01 | 0.00 |
|  | Antihypertensive drugs | 80.91 | 80.38 | 0.06 | 0.01 |
|  | Antiplatelet drugs | 25.09 | 25.58 | -0.03 | -0.01 |
|  | Corticosteroids (systemic use) | 14.83 | 15.07 | -0.03 | -0.01 |
|  | Fondaparinux | 0.78 | 0.68 | 0.02 | 0.01 |
|  | Heparins | 26.45 | 22.99 | 0.37 | 0.07 |
|  | Insulin | 10.97 | 10.55 | 0.04 | 0.01 |
|  | Lipid-lowering agents | 47.55 | 46.3 | 0.09 | 0.03 |
|  | NSAIDs | 39.98 | 40.85 | -0.05 | -0.02 |
|  | Oral anti-diabetic drugs | 20.11 | 20.13 | 0.01 | 0.00 |
|  | Peptic Ulcer/Reflux disease | 49.81 | 50.28 | -0.04 | -0.01 |
|  | SSRIs | 5.37 | 5.61 | -0.04 | -0.01 |
| Comorbidities (%) | Alcohol abuse/addiction | 2.34 | 2.41 | -0.02 | -0.01 |
|  | Bleeding extracerebral | 5.55 | 5.1 | 0.05 | 0.02 |
|  | Bleeding gastrointestinal | 5.85 | 5.66 | 0.02 | 0.01 |
|  | Bleeding intracerebral | 0.44 | 0.48 | -0.02 | -0.01 |
|  | Cancer | 19.71 | 19.73 | 0.00 | 0.00 |
|  | Coagulopathy | 1.82 | 1.62 | 0.03 | 0.01 |
|  | Congestive Heart Failure | 28.94 | 27.1 | 0.12 | 0.04 |
|  | COPD | 16.05 | 15.78 | 0.02 | 0.01 |
|  | Coronary Heart Disease | 40.6 | 38.55 | 0.13 | 0.04 |
|  | Diabetes | 38.65 | 37.88 | 0.05 | 0.02 |
|  | Diverticulitis | 10.66 | 10.79 | -0.01 | 0.00 |
|  | Embolism systemic | 0.8 | 0.69 | 0.03 | 0.01 |
|  | Embolism venous | 5.14 | 4.72 | 0.05 | 0.02 |
|  | Esophageal varices | 0.22 | 0.21 | 0.00 | 0.00 |
|  | Hypertension | 88.14 | 87.77 | 0.05 | 0.01 |
|  | Ischemic Stroke | 8.58 | 8.41 | 0.01 | 0.01 |
|  | Liver disease | 13.44 | 13.26 | 0.02 | 0.01 |
|  | Nicotin use/dependence | 4.46 | 4.52 | -0.01 | 0.00 |
|  | Renal disease | 14.87 | 13.25 | 0.12 | 0.04 |
|  | Upper gastrointestinal system | 26.04 | 26.3 | -0.03 | -0.01 |
|  | Vascular disease | 21.96 | 21.46 | 0.04 | 0.01 |
|  | Vascular dementia | 1.85 | 1.99 | -0.05 | -0.01 |
|  | Venous malformation | 1.86 | 1.63 | 0.04 | 0.02 |

#### Mortality Population

Table S18

| **Patient characteristics mortality population** | | **VKA**  (n=347351) | **DOAC**  (n=347351) | **Smd** | |
| --- | --- | --- | --- | --- | --- |
|  |  |  |  | Before | After |
| Age | Mean(± SD) | 75.76(±8.85) | 75.7(±9.84) | 0.03 | 0.01 |
|  | Median | 77 | 77 |  |  |
| Age distribution (%) | 18-36 | 0.09 | 0.19 |  |  |
|  | 37-54 | 2.16 | 3.22 |  |  |
|  | 55-72 | 27.56 | 27.42 |  |  |
|  | 73+ | 70.19 | 69.17 |  |  |
| Female Sex (%) |  | 52 | 52.92 | -0.07 | -0.02 |
| CHA_2_DS_2_-VASc Score | Mean(± SD) | 4.45(±1.7) | 4.39(±1.77) | 0.11 | 0.04 |
|  | Median | 4 | 4 |  |  |
| CHA_2_DS_2_-VASc Score distribution (%) | 0-1 | 3.78 | 5.3 |  |  |
|  | 2-3 | 25.18 | 25.26 |  |  |
|  | 4-5 | 44.76 | 43.23 |  |  |
|  | 6-7 | 22.32 | 22.03 |  |  |
|  | 8-9 | 3.96 | 4.18 |  |  |
| Charlson Comorbidity  Index | Mean(± SD) | 2.92(±2.63) | 2.84(±2.63) | 0.09 | 0.03 |
|  | Median | 2 | 2 |  |  |
| Charlson Comorbidity  Index distribution (%) | 0-4 | 76.81 | 77.68 |  |  |
|  | 5-9 | 20.88 | 20 |  |  |
|  | 10-15 | 2.22 | 2.24 |  |  |
|  | 15+ | 0.15 | 0.14 |  |  |
| Number of distinct  prescriptions (ATCs) | Mean(± SD) | 12.5(±5.72) | 12.43(±5.78) | 0.05 | 0.01 |
|  | Median | 12 | 11 |  |  |
| Number of  prescriptions (ATCs) | Mean(± SD) | 35.41(±21.15) | 34.88(±21.43) | 0.08 | 0.02 |
|  | Median | 31 | 30 |  |  |
| Number of diagnoses  (ICD-Codes) | Mean(± SD) | 28.21(±15.38) | 27.95(±15.53) | 0.04 | 0.02 |
|  | Median | 26 | 25 |  |  |
| Prescribed Medicines  (%) | Antiarrhythmic agents | 90.51 | 90.44 | 0.01 | 0.00 |
|  | Antihypertensive drugs | 80.94 | 80.42 | 0.06 | 0.01 |
|  | Antiplatelet drugs | 25.08 | 25.56 | -0.03 | -0.01 |
|  | Corticosteroids (systemic use) | 14.79 | 15.04 | -0.03 | -0.01 |
|  | Fondaparinux | 0.78 | 0.68 | 0.02 | 0.01 |
|  | Heparins | 27.15 | 23.54 | 0.36 | 0.08 |
|  | Insulin | 10.93 | 10.56 | 0.04 | 0.01 |
|  | Lipid-lowering agents | 47.59 | 46.31 | 0.09 | 0.03 |
|  | NSAIDs | 39.99 | 40.76 | -0.05 | -0.02 |
|  | Oral anti-diabetic drugs | 20.1 | 20.14 | 0.00 | 0.00 |
|  | Peptic Ulcer/Reflux disease | 49.88 | 50.3 | -0.04 | -0.01 |
|  | SSRIs | 5.37 | 5.58 | -0.04 | -0.01 |
| Comorbidities (%) | Alcohol abuse/addiction | 2.33 | 2.44 | -0.02 | -0.01 |
|  | Bleeding extracerebral | 5.52 | 5.14 | 0.05 | 0.02 |
|  | Bleeding gastrointestinal | 5.85 | 5.69 | 0.02 | 0.01 |
|  | Bleeding intracerebral | 0.45 | 0.46 | -0.02 | 0.00 |
|  | Cancer | 19.74 | 19.79 | 0.00 | 0.00 |
|  | Coagulopathy | 1.82 | 1.63 | 0.03 | 0.01 |
|  | Congestive Heart Failure | 28.95 | 27.18 | 0.12 | 0.04 |
|  | COPD | 16.02 | 15.82 | 0.02 | 0.01 |
|  | Coronary Heart Disease | 40.66 | 38.71 | 0.13 | 0.04 |
|  | Diabetes | 38.59 | 37.9 | 0.05 | 0.01 |
|  | Diverticulitis | 10.64 | 10.76 | -0.01 | 0.00 |
|  | Embolism systemic | 0.81 | 0.7 | 0.03 | 0.01 |
|  | Embolism venous | 5.15 | 4.77 | 0.05 | 0.02 |
|  | Esophageal varices | 0.21 | 0.21 | 0.00 | 0.00 |
|  | Hypertension | 88.13 | 87.83 | 0.05 | 0.01 |
|  | Ischemic Stroke | 8.58 | 8.41 | 0.01 | 0.01 |
|  | Liver disease | 13.44 | 13.29 | 0.02 | 0.00 |
|  | Nicotin use/dependence | 4.44 | 4.5 | -0.01 | 0.00 |
|  | Renal disease | 14.87 | 13.31 | 0.12 | 0.04 |
|  | Upper gastrointestinal system | 26.02 | 26.28 | -0.03 | -0.01 |
|  | Vascular disease | 22.01 | 21.46 | 0.04 | 0.01 |
|  | Vascular dementia | 1.86 | 1.98 | -0.05 | -0.01 |
|  | Venous malformation | 1.85 | 1.65 | 0.04 | 0.01 |

## Cumulative incidence

Table S19 Cumulative incidence in VKA patients Number at risk and censored (cens) patients

| Cohort | Stroke | | Embolism | | TIA | | Bleeding | | Mortality | |
| --- | --- | --- | --- | --- | --- | --- | --- | --- | --- | --- |
| Time | risk | cens | risk | cens | risk | cens | risk | cens | risk | cens |
| 1 | 347240 | 101757 | 347297 | 103117 | 347247 | 102889 | 346343 | 97882 | 347351 | 93211 |
| 2 | 241655 | 30104 | 243629 | 30712 | 243048 | 30643 | 233817 | 27798 | 247465 | 27428 |
| 3 | 209551 | 48355 | 212610 | 49544 | 211606 | 49274 | 197013 | 43834 | 214040 | 46336 |
| 4 | 159722 | 22748 | 162806 | 23518 | 161616 | 23237 | 145938 | 19964 | 164570 | 21686 |
| 5 | 135927 | 23027 | 139148 | 23920 | 137947 | 23592 | 120890 | 19903 | 140385 | 22270 |
| 6 | 112112 | 15293 | 115125 | 15791 | 113981 | 15703 | 97240 | 12826 | 116384 | 14718 |
| 7 | 96291 | 15890 | 99248 | 16570 | 97993 | 16244 | 81533 | 13149 | 100255 | 15604 |
| 8 | 79934 | 10485 | 82617 | 10897 | 81518 | 10773 | 66154 | 8346 | 83471 | 10118 |
| 9 | 69115 | 9872 | 71645 | 10259 | 70588 | 10110 | 55961 | 7807 | 72395 | 9751 |
| 10 | 58981 | 8395 | 61340 | 8832 | 60331 | 8690 | 46703 | 6470 | 61896 | 8279 |
| 11 | 50363 | 8537 | 52474 | 8995 | 51539 | 8786 | 39032 | 6476 | 52972 | 8484 |
| 12 | 41653 | 6172 | 43457 | 6504 | 42667 | 6378 | 31563 | 4527 | 43930 | 6105 |
| 13 | 35346 | 5392 | 36926 | 5673 | 36212 | 5555 | 26218 | 3889 | 37339 | 5384 |
| 14 | 29845 | 5615 | 31233 | 5975 | 30598 | 5810 | 21678 | 4021 | 31591 | 5609 |
| 15 | 24143 | 5774 | 25244 | 6059 | 24741 | 5976 | 17188 | 4019 | 25634 | 5910 |
| 16 | 18300 | 3567 | 19169 | 3801 | 18730 | 3671 | 12775 | 2465 | 19459 | 3651 |
| 17 | 14680 | 3291 | 15360 | 3457 | 15026 | 3365 | 10030 | 2183 | 15606 | 3355 |
| 18 | 11355 | 3530 | 11897 | 3704 | 11640 | 3597 | 7648 | 2300 | 12098 | 3620 |
| 19 | 7794 | 4144 | 8184 | 4315 | 8032 | 4248 | 5182 | 2716 | 8354 | 4334 |
| 20 | 3628 | 2111 | 3866 | 2242 | 3772 | 2198 | 2366 | 1342 | 3930 | 2238 |
| 21 | 1513 | 1511 | 1621 | 1621 | 1571 | 1568 | 972 | 951 | 1648 | 1634 |

Table S20 Cumulative incidence in DOAC patients Number at risk and censored (cens) patients

| Cohort | Stroke | | Embolism | | TIA | | Bleeding | | Mortality | |
| --- | --- | --- | --- | --- | --- | --- | --- | --- | --- | --- |
| Time | risk | cens | risk | cens | risk | cens | risk | cens | risk | cens |
| 1 | 347240 | 38901 | 347297 | 39135 | 347247 | 38968 | 346343 | 37654 | 347351 | 30794 |
| 2 | 303332 | 24532 | 307753 | 24940 | 306867 | 24934 | 295452 | 22761 | 309145 | 18934 |
| 3 | 275764 | 37876 | 282532 | 39161 | 280916 | 38741 | 262934 | 35250 | 283794 | 33938 |
| 4 | 235404 | 30684 | 243137 | 31899 | 241243 | 31505 | 219250 | 27750 | 244619 | 27809 |
| 5 | 202742 | 25391 | 211066 | 26598 | 209005 | 26359 | 185028 | 22851 | 212466 | 23340 |
| 6 | 175841 | 23872 | 184331 | 25066 | 182056 | 24598 | 156944 | 20782 | 185553 | 22139 |
| 7 | 150809 | 24060 | 159147 | 25411 | 156965 | 25077 | 132128 | 20595 | 160287 | 22843 |
| 8 | 125804 | 18787 | 133643 | 19975 | 131454 | 19603 | 108195 | 15790 | 134678 | 17807 |
| 9 | 106326 | 15778 | 113584 | 16928 | 111549 | 16561 | 89884 | 13005 | 114666 | 15190 |
| 10 | 89971 | 14521 | 96596 | 15680 | 94731 | 15496 | 74705 | 11937 | 97572 | 14247 |
| 11 | 74991 | 14851 | 80865 | 15965 | 79058 | 15567 | 61111 | 11905 | 81659 | 14731 |
| 12 | 59736 | 10952 | 64854 | 11889 | 63324 | 11588 | 47827 | 8620 | 65525 | 10914 |
| 13 | 48473 | 9583 | 52934 | 10511 | 51624 | 10316 | 38122 | 7392 | 53449 | 9784 |
| 14 | 38644 | 9471 | 42395 | 10414 | 41202 | 10041 | 29906 | 7211 | 42744 | 9711 |
| 15 | 29007 | 8849 | 31961 | 9724 | 31073 | 9447 | 22103 | 6577 | 32322 | 9256 |
| 16 | 20010 | 5532 | 22224 | 6164 | 21561 | 5892 | 15038 | 4095 | 22481 | 5842 |
| 17 | 14382 | 4482 | 16050 | 5025 | 15633 | 4850 | 10619 | 3282 | 16253 | 4773 |
| 18 | 9833 | 3927 | 11014 | 4428 | 10746 | 4344 | 7128 | 2857 | 11166 | 4281 |
| 19 | 5856 | 3338 | 6582 | 3705 | 6387 | 3614 | 4122 | 2278 | 6664 | 3623 |
| 20 | 2491 | 1557 | 2874 | 1810 | 2762 | 1746 | 1740 | 1092 | 2899 | 1754 |
| 21 | 924 | 918 | 1064 | 1064 | 1015 | 1015 | 615 | 598 | 1086 | 1056 |

## Heparin related Analysis

36.44% of VKA users but only 18.02% - 19.53% of DOAC users were treated with a heparin in the quarter with the 1^st^ OAC prescription or the 4 quarters before.

VKA and DOAC users who received heparin treatment did not differ considerably in comorbidities that would justify a heparin therapy. The only differences greater 3% were observed in coronary heart disease (4.4%) and renal dysfunction (3.4%), both of which VKA users were more often affected by.

Further differences in the timing of the prescription were examined, which would provide evidence for a hypothesis based on pharmaceutical reasons. Besides procoagulatory factors II, VII, IX and X also the anticoagulation factors Protein C and S are dependent on Vitamin-K. Because the half-life of the Proteins C and S is much shorter than of the Vitamin-K-dependent procoagulatory factors there are less anticoagulatory factors than procoagulatory factors available during the first days of VKA therapy initiation. This leads to a procoagulatory phase and is recommended to be compensated by an anticoagulatory therapy with heparin [40] depending on the individual risk assessment. In case heparin was most often prescribed at the same time like VKAs this might indicate a concomitant therapy during therapy initiation.

The analysis showed that relative to the total number of heparin prescriptions per OAC group, VKA users showed more often heparin prescriptions in the same quarter like the first OAC prescription, than DOAC users (Figure S3a). DOAC users on the other hand were prescribed heparins relatively more often in the 1-4 quarters before the first OAC prescription. In a more detailed analysis of heparin prescriptions in the quarter of the first OAC prescription, this finding was further dissected (Figure S3b). This revealed that in case of VKA the most heparin prescriptions were made at the same date like the first VKA prescription (38.2%), while in DOAC users the heparin prescription preceded the first DOAC prescription most often (60.3%). Roughly, the same number of VKA (27.0%) und DOAC (21.7%) users got a heparin prescription after the first OAC prescription. This supports the hypothesis that the greater proportion of heparin prescriptions in VKA users compared to DOAC users is caused by a concomitant therapy during therapy initiation. It can therefore be assumed that the heparin treatment is a short-term therapy, which does not affect the final study outcome. However, to investigate the influence of heparin therapy reliably, we excluded all patients with heparin therapy from the stroke and bleeding cohorts, conducted new matchings and calculated new hazard ratios. This revealed almost identical results with maximal changes of 0.02 points in hazard ratios (Table S21). These results further support the hypotheses that the initial heparin therapy in VKA patients is not relevant in the further course of the disease.

Figure S3 a) Heparin prescriptions relative to all heparin prescriptions within VKA or DOAC patients during 1-4 quarters before or in the same quarter like the first OAC prescription; b) Heparin prescriptions relative to all heparin prescriptions within VKA or DOAC patients in the same quarter like the first OAC prescription. Heparin_OAC: First heparin, then OAC; OAC_Heparin: First OAC, then heparin.

Table S21: Hazard ratios heparin related analysis

|  | **All patients included** | | **Patients with heparin excluded** | |
| --- | --- | --- | --- | --- |
| Events | Adjusted HR  (95% CI) | p value | Adjusted HR  (95% CI) | p value |
| Stroke | 1.32 (1.29-1.35) | <.001 | 1.33 (1.29-1.36) | <.001 |
| Bleeding | 0.89 (0.88-0.90) | <.001 | 0.91 (0.89-0.92) | <.001 |

## Separate DOAC Analysis

### Patient characteristics before matching: Stroke Population

Table S22

| **Patient characteristics before matching**  **Stroke populations** | | **VKA**  (n=405437) | **Dabigatran**  (n=53057) | **Rivaroxaban**  (n=228609) | **Apixaban**  (n=131751) | **Edoxaban**  (n=14276) |
| --- | --- | --- | --- | --- | --- | --- |
| Age | Mean  (± SD) | 75.71  (±8.82) | 74.44  (±9.98) | 75.04  (±10.07) | 76.64  (±9.80) | 75.48  (±9.26) |
|  | Median | 77.00 | 76.00 | 76.00 | 78.00 | 76.00 |
| Age distribution  (%) | 18-36 | 0.09 | 0.22 | 0.23 | 0.15 | 0.11 |
|  | 37-54 | 2.17 | 4.01 | 3.72 | 2.72 | 2.65 |
|  | 55-72 | 27.65 | 31.80 | 29.50 | 24.84 | 28.96 |
|  | 73+ | 70.09 | 63.98 | 66.55 | 72.28 | 68.28 |
| Female Sex (%) |  | 51.10 | 51.96 | 54.43 | 56.09 | 54.68 |
| CHA_2_DS_2_-VASc  Score | Mean  (± SD) | 4.48  (±1.71) | 4.15  (±1.78) | 4.21  (±1.78) | 4.44  (±1.75) | 4.28  (±1.71) |
|  | Median | 4.00 | 4.00 | 4.00 | 4.00 | 4.00 |
| CHA_2_DS_2_-VASc  Score distribution (%) | 0-1 | 3.71 | 6.92 | 6.48 | 4.72 | 4.83 |
|  | 2-3 | 24.73 | 28.99 | 27.94 | 24.31 | 27.33 |
|  | 4-5 | 44.59 | 41.83 | 42.41 | 44.18 | 44.91 |
|  | 6-7 | 22.77 | 19.02 | 19.67 | 22.49 | 19.70 |
|  | 8-9 | 4.19 | 3.23 | 3.51 | 4.30 | 3.24 |
| Charlson Comorbidity Index | Mean  (± SD) | 2.98  (±2.66) | 2.51  (±2.44) | 2.67  (±2.57) | 2.95  (±2.69) | 2.80  (±2.63) |
|  | Median | 2.00 | 2.00 | 2.00 | 2.00 | 2.00 |
| Charlson Comorbidity Index distribution (%) | 0-4 | 75.97 | 82.02 | 79.84 | 76.21 | 78.12 |
|  | 5-9 | 21.58 | 16.37 | 18.07 | 21.23 | 19.52 |
|  | 10-15 | 2.35 | 1.57 | 2.02 | 2.45 | 2.29 |
|  | 15+ | 0.17 | 0.08 | 0.12 | 0.16 | 0.15 |
| No. of distinct prescriptions (ATCs) | Mean  (± SD) | 12.68  (±5.77) | 12.06  (±5.57) | 12.31  (±5.77) | 12.76  (±5.83) | 11.92  (±5.60) |
|  | Median | 12.00 | 11.00 | 11.00 | 12.00 | 11.00 |
| No. of prescriptions (ATCs) | Mean  (± SD) | 36.03  (±21.54) | 32.97  (±19.98) | 33.79  (±21.00) | 35.90  (±21.64) | 33.05  (±20.29) |
|  | Median | 31.00 | 29.00 | 29.00 | 31.00 | 28.00 |
| No. of diagnoses  (ICD-Codes) | Mean  (± SD) | 28.49  (±15.51) | 26.77  (±14.96) | 27.48  (±15.36) | 28.85  (±15.91) | 29.03  (±15.74) |
|  | Median | 26.00 | 24.00 | 25.00 | 26.00 | 26.00 |
| Prescribed Medicines (%) | Antiarrhythmic agents | 90.65 | 90.16 | 90.14 | 90.83 | 89.28 |
|  | Antihypertensive drugs | 81.38 | 78.60 | 78.45 | 80.14 | 78.75 |
|  | Antiplatelet drugs | 24.98 | 26.48 | 24.78 | 28.29 | 24.31 |
|  | Corticosteroids | 14.76 | 14.68 | 15.81 | 15.95 | 14.89 |
|  | Fondaparinux | 0.80 | 0.66 | 0.61 | 0.52 | 0.50 |
|  | Heparins | 36.44 | 19.53 | 19.20 | 18.02 | 19.52 |
|  | Insulin | 11.25 | 9.04 | 9.86 | 10.91 | 9.92 |
|  | Lipid-lowering agents | 48.47 | 45.77 | 41.91 | 46.97 | 43.39 |
|  | NSAIDs | 39.76 | 42.95 | 43.13 | 40.86 | 42.51 |
|  | Oral anti-diabetic drugs | 20.14 | 19.59 | 19.93 | 19.98 | 20.30 |
|  | Peptic Ulcer/Reflux disease | 50.04 | 50.00 | 51.08 | 53.85 | 49.28 |
|  | SSRIs | 5.21 | 6.04 | 6.00 | 6.47 | 5.27 |
|  | Alcohol abuse/addiction | 2.28 | 2.65 | 2.69 | 2.50 | 2.36 |
| Comorbidities (%) | Bleeding extracerebral | 5.81 | 4.62 | 4.63 | 4.87 | 4.64 |
|  | Bleeding gastrointestinal | 6.04 | 5.61 | 5.46 | 5.73 | 5.55 |
|  | Bleeding intracerebral | 0.42 | 0.68 | 0.48 | 0.61 | 0.49 |
|  | Cancer | 19.96 | 18.80 | 19.86 | 20.72 | 20.86 |
|  | Coagulopathy | 1.94 | 1.41 | 1.39 | 1.60 | 1.51 |
|  | Congestive Heart Failure | 29.82 | 22.65 | 24.00 | 26.19 | 24.05 |
|  | COPD | 16.20 | 14.51 | 15.50 | 15.79 | 15.22 |
|  | Coronary Heart Disease | 41.73 | 34.83 | 34.49 | 36.92 | 35.75 |
|  | Diabetes | 39.10 | 35.41 | 36.40 | 37.50 | 36.68 |
|  | Diverticulitis | 10.67 | 10.58 | 10.72 | 11.82 | 12.35 |
|  | Embolism systemic | 0.88 | 0.56 | 0.61 | 0.61 | 0.61 |
|  | Embolism venous | 5.48 | 3.71 | 4.53 | 4.17 | 4.38 |
|  | Esophageal varices | 0.21 | 0.24 | 0.21 | 0.24 | 0.23 |
|  | Hypertension | 88.40 | 86.06 | 86.48 | 87.80 | 88.05 |
|  | Ischemic Stroke | 8.69 | 9.33 | 7.68 | 8.80 | 6.93 |
|  | Liver disease | 13.61 | 12.91 | 12.91 | 13.35 | 13.46 |
|  | Nicotin use/dependence | 4.44 | 4.49 | 4.75 | 4.60 | 4.38 |
|  | Renal disease | 15.91 | 8.75 | 10.83 | 13.56 | 12.45 |
|  | Upper gastrointestinal system | 25.99 | 26.28 | 27.04 | 27.59 | 28.19 |
|  | Vascular disease | 22.43 | 19.43 | 19.85 | 22.51 | 21.43 |
|  | Vascular dementia | 1.73 | 1.91 | 2.24 | 2.78 | 2.28 |
|  | Venous malformation | 1.99 | 1.47 | 1.35 | 1.53 | 1.46 |

### Sensitivity Analysis I and II

7,629 patients with rivaroxaban or dabigatran prescriptions were treated with doses that are not explicitly approved for stroke prevention in AF patients. In the total number of dabigatran and rivaroxaban patients, they accounted for about 2.7%. It is likely that very low doses of dabigatran and rivaroxaban are prescribed for similar reasons than low doses of other DOACs, which are explicitly approved for patients with AF. For instance, the lowest dose of edoxaban (15mg) is not recommended as monotherapy but only in case the patient is switching from edoxaban 30mg to VKA and in combination with an appropriate VKA dose [38]. Further, the lowest dose of rivaroxaban (2.5mg) is approved as concomitant therapy in patients who are prescribed acetylsalicylic acid after an acute coronary syndrome (ACS) as well as with coronary heart disease or peripheral arterial occlusive disease [39]. Finally, it might be expected that patients with very high risk of bleeding but low risk of stroke or severely limited renal or liver function be treated with the lowest available dose.

Table S23 Results of sensitivity analysis I and II for joint DOACs, dabigatran and rivaroxaban Sensitivity analysis I includes only doses approved for stroke prevention in AF patients; Sensitivity analysis II includes only patients without an alternative diagnosis than AF, which might also have led to a DOAC prescription

| **Events** | **Sensitivity analysis I** | |  | **Sensitivity analysis II** | |
| --- | --- | --- | --- | --- | --- |
|  | **Adjusted HR (95%CI)** | **p value** |  | **Adjusted HR (95%CI)** | **p value** |
|  | Joint DOAC | | | | |
| Stroke | 1.31 (1.28-1.34) | <0.001 |  | 1.32 (1.29-1.36) | <0.001 |
| TIA | 1.07 (1.04-1.12) | <0.001 |  | 1.10 (1.06-1.14) | <0.001 |
| Embolism (systemic) | 0.76 (0.71-0.81) | <0.001 |  | 0.79 (0.74-0.85) | <0.001 |
| Bleeding | 0.88 (0.87-0.89) | <0.001 |  | 0.89 (0.88-0.90) | <0.001 |
|  | Dabigatran | | | | |
| Stroke | 1.93(1.82-2.03) | <0.001 |  | 1.92(1.82-2.03) | <0.001 |
| TIA | 1.33(1.22-1.45) | <0.001 |  | 1.32(1.21-1.44) | <0.001 |
| Embolism (systemic) | 0.92(0.78-1.09) | 0.335 |  | 0.96(0.81-1.14) | 0.626 |
| Bleeding | 0.85(0.83-0.88) | <0.001 |  | 0.85(0.83-0.88) | <0.001 |
|  | Rivaroxaban | | | | |
| Stroke | 1.12(1.09-1.15) | <0.001 |  | 1.13(1.10-1.16) | <0.001 |
| TIA | 1.05(1.00-1.10) | 0.036 |  | 1.06(1.01-1.11) | 0.022 |
| Embolism (systemic) | 0.80(0.74-0.87) | <0.001 |  | 0.83(0.77-0.91) | <0.001 |
| Bleeding | 1.02(1.00-1.03) | 0.016 |  | 1.03(1.01-1.04) | <0.001 |

Table S24 Results of sensitivity analysis II for edoxaban and apixaban Sensitivity analysis II includes only patients without an alternative diagnosis than AF, which might also have led to a DOAC prescription

|  | **Sensitivity analysis II** | | | | |
| --- | --- | --- | --- | --- | --- |
|  | **Edoxaban** | |  | **Apixaban** | |
| **Events** | **Adjusted HR (95%CI)** | **p value** |  | **Adjusted HR (95%CI)** | **p value** |
| Stroke | 0.86(0.72-1.04) | 0.117 |  | 1.52(1.46-1.58) | <0.001 |
| TIA | 0.70(0.52-0.95) | 0.020 |  | 1.15(1.08-1.23) | <0.001 |
| Embolism (systemic) | 0.30(0.17-0.53) | <0.001 |  | 0.77(0.68-0.87) | <0.001 |
| Bleeding | 0.74(0.67-0.81) | <0.001 |  | 0.71(0.70-0.73) | <0.001 |
